# In-Silico Screening of Novel Synthesized Thienopyrimidines Targeting Fms Related Receptor Tyrosine Kinase-3 and Their In-Vitro Biological Evaluation

**DOI:** 10.3390/ph15020170

**Published:** 2022-01-29

**Authors:** Elshaymaa I. Elmongy, Najla Altwaijry, Nashwah G. M. Attallah, Manal Mubarak AlKahtani, Hanan Ali Henidi

**Affiliations:** 1 Department of Pharmaceutical Sciences, College of Pharmacy, Princess Nourah bint Abdulrahman University, Riyadh 84428, Saudi Arabia; naaltwaijry@pnu.edu.sa (N.A.); ngmohamed@pnu.edu.sa (N.G.M.A.); 2 Department of Pharmaceutical Chemistry, Faculty of Pharmacy, Helwan University, Ain Helwan, Cairo 11795, Egypt; 3 Egyptian Drug Authority (EDA) (Previously NODCAR), Giza 8655, Egypt; 4 Research Department, Health Sciences Research Center, Princess Nourah Bint Abdulrahman University, Riyadh 84428, Saudi Arabia; mamalkahtani@pnu.edu.sa (M.M.A.); hahenidi@pnu.edu.sa (H.A.H.)

**Keywords:** thienopyrimidines, selectivity index, in-silico screening, tyrosine kinase enzyme

## Abstract

The present investigation describes the design strategy and synthesis of novel thienopyrimidine compounds in addition to their anticancer activity targeting tyrosine kinase FLT3 enzyme. The synthesized compounds were subjected to a cytotoxic study where compounds **9a** and **9b** showed the most potent cytotoxicity against HT-29, HepG-2, and MCF-7 cell lines reflected by their IC_50_ values for **9a** (1.21 ± 0.34, 6.62 ± 0.7 and 7.2 ± 1.9 μM), for **9b** (0.85 ± 0.16, 9.11 ± 0.3 and 16.26 ± 2.3 μM) and better than that of reference standard which recorded (1.4 ± 1.16, 13.915 ± 2.2, and 8.43 ± 0.5 μM), respectively. Compounds’ selectivity to malignant cells was determined using selectivity assay, interestingly, all the tested compounds demonstrated an excellent selectivity index (SI) range from 20.2 to 99.7. Target in-silico prediction revealed the FLT3 kinase enzyme was the kinase enzyme of highest probability. Molecular docking studies were performed on the prepared compounds which showed promising binding affinity for FLT3 kinase enzyme and the main interactions between the synthesized ligands and kinase active site were similar to those between the co-crystallized ligand and the receptor. Further biological exploration was performed using in-vitro FLT3 kinase enzyme inhibition assay. The results showed that the 2-morpholinoacetamido derivative **10a** exhibited highest FLT3 inhibitory activity among the tested compounds followed by compound **9a** then **12**. Pharmacokinetic assessment disclosed that all the investigated compounds were considered as “drug-like” molecules with promising bioavailability.

## 1. Introduction

Protein kinases (PKs) are protein phosphorylating enzymes, responsible for transducing signals from the cell membrane into the cell’s interior, which regulates most aspects of proper cellular activity. The molecular basis of many malignancies and various signs of cardiovascular illness, such as hypertrophy, angiogenesis, and atherogenesis, is pathological malfunction of protein kinase signaling pathways. Protein kinases are becoming appealing targets for drug discovery due to their participation in such a wide range of disease conditions [1,2,3,4]. 

**Figure 1:**
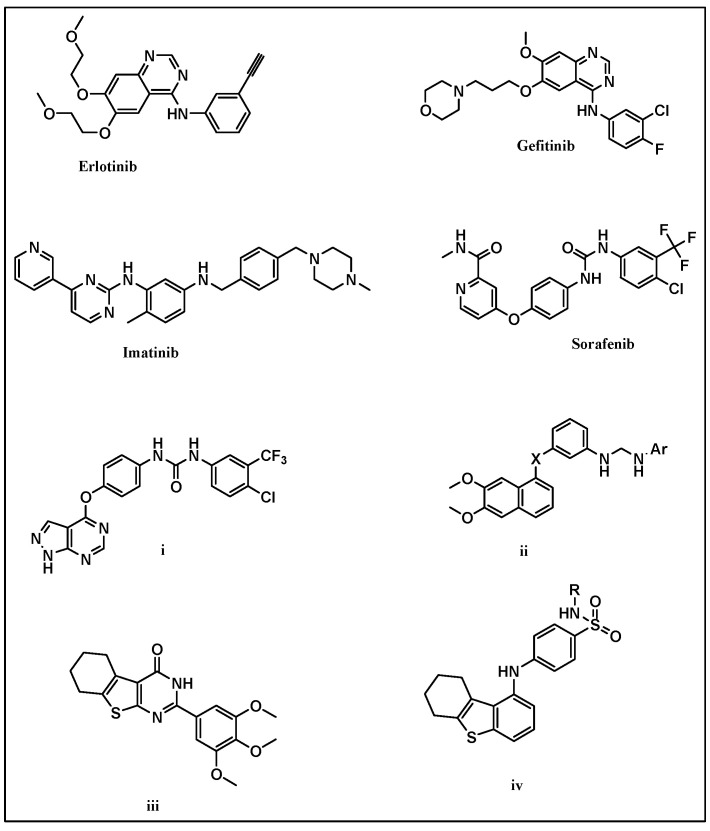
Examples of reported kinase inhibitors.

Tyrosine kinases phosphorylate specific amino acids on substrate enzymes, which affects cell growth due to signal transduction downstream. Constitutive activation or inhibition of TKs can lead to abnormal signal cascades as well, resulting in malignancy, apoptosis, and cell death [1]. Accordingly, tyrosine kinase inhibitors (TKIs) can prevent mutant or malfunctioning TKs from acting abnormally by inhibiting these initial signals. Imatinib is a tyrosine kinase inhibitor that brought interest to TKIs when approved by the FDA, Figure 1. Since the introduction of Imatinib, and due to tyrosine kinases’ critical roles in cellular signaling, the application of TKIs particularly for cancer treatment has become an attractive target [2,3].

Fms-like tyrosine kinase 3 (FLT3) is one of the interesting candidates of TKs that is important to maintain normal stem cell development as it supports hematopoietic stem cells proliferation and survival when activated by its ligand (FLT3L) [4,5]. Reviewing the literature revealed that thieno[2,3-*d*]pyrimidine derivatives may act as potent FLT3 inhibitors [6]. Thieno[2,3-*d*]pyrimidine ring system is considered as an attractive scaffold with various biological activities [7,8,9,10]. Moreover, in reference to the reported anticancer drugs gefitinib and erlotinib, thienopyrimidine ring scaffold is considered as a bioisostere of their core nucleus 4-aminoquinazoline [6], Figure 1.

Sorafenib is a diaryl urea derivative that acts as a multityrosine kinase inhibitor [11]. By modification of the sorafenib structure, several analogues have been synthesized to improve its cytotoxic and antiangiogenic activities. These modifications included replacement of urea with thiourea [12], with different ring scaffolds such as pyrazolo pyrimidine **i** and quinazoline **ii** [13,14]. In addition, several papers reported that the introduction of a lipophilic cycloalkyl ring to the chemical scaffold of thieno[2,3-*d]*pyrimidines as shown in compounds (**iii** and **iv**) enhances the anticancer activity [15,16,17], Figure 1.

Inspired by these findings, herein we designed a new series of cyclohexylthieno[2,3-*d]*pyrimidines in an attempt to synthesize anticancer compounds targeting tyrosine kinases with a promising inhibitory profile.

### Design Strategy

The design strategy was based on introducing cyclohexylthieno[2,3-*d*]pyrimidine as a core structure in reference to literature reporting the improved anticancer activity upon introducing a lipophilic cycloalkyl ring to the fused thieno[2,3-*d]*pyrimidine ring system [15,16,17]. Incorporating various functional groups at position 2 was then done. These introduced groups had been previously reported in compounds with promising anticancer activity and especially kinase inhibition profile such as quizartinib and sorafenib. These modifications were also supported by previously reported research on compounds bearing a thieno[2,3-*d*]pyrimidine nucleus and having significant kinase inhibitory activity [6,18,19].

**Figure 2:**
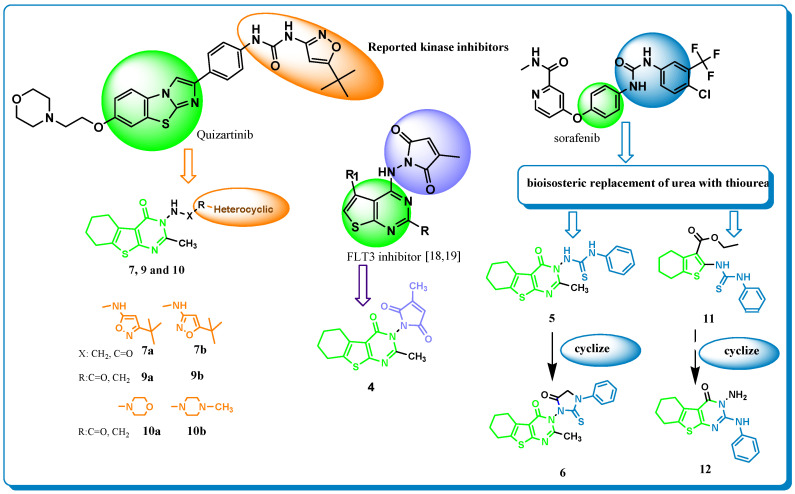
Design strategy for the synthesized compounds in relation to reported kinase inhibitors quizartinib and sorafenib.

Groups introduced to the prepared nucleus as the methyl pyrrole-2,5-dione moiety in addition to the primary amine “3-*tert*-butyl isoxazal-5-yl amine” which is presented in “quizartinib” kinase inhibitor, were cited in literature [6,18,19], also in a step for comparing the kinase inhibition results attributed to the isosteric replacement, 5-*tert*-butyl isoxazal-3-yl amine was introduced, Figure 1. Replacing of the incorporated primary amine moieties with secondary amines “morpholine and *N*-methyl piperazine” was performed, aiming to test the effect of replacement on biological activity, Figure 2.

Moreover, referring to sorafenib as a reported multityrosine kinase inhibitor bearing a diaryl urea moiety, several analogues have been synthesized aiming to improve the angiogenic activities [12]. These modifications include replacement of urea with thiourea or with other scaffolds such as pyrazolo pyrimidine **i** and quinazoline **ii** [13,14], which prompted our interest to incorporate thiourea as a bio-isosteric replacement of urea to either thienopyrimidine nucleus as in compound **11** or the thiophene core **5** which is also a bioisostere of the benzene ring of sorafenib. Cyclization of compounds **5** and **11** was performed resulting in compounds **6** and **12,** respectively, in order to explore the effect of rigidification on anticancer and kinase inhibition performance, Figure 2.

## 2. Results and Discussion

### 2.1. Chemistry

**Scheme 1:**
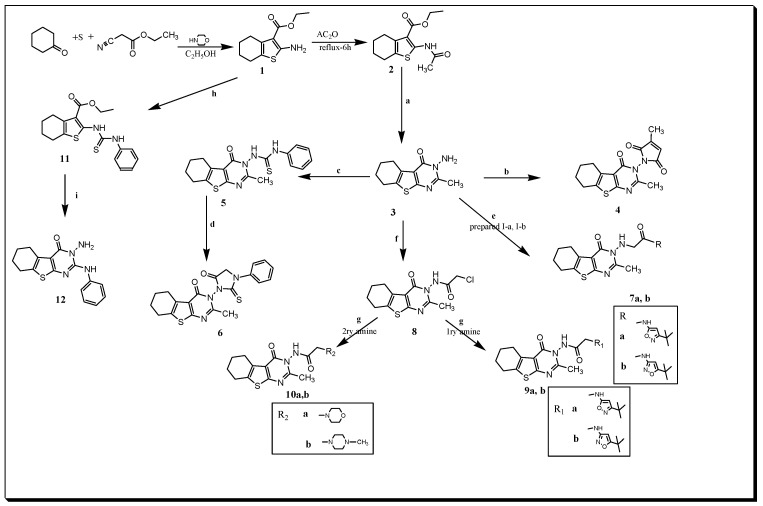
Synthesis pathways for the designed compounds **1**–**12**. a: NH_2_NH_2_.H_2_O, ethanol, 130^o^; b: citraconic anhydride, CHCl_3_, reflux, 11 h; c: PhNCS, DCMreflux, 14 h; d: ClCOCH_2_Cl, dry benzene, TEA, reflux 11 h; e: TEA, heat 80^o^, 12 h; f: ClCOCH_2_Cl, gentle reflux 110^o^,4h; g: DCM, TEA, reflux 11h; h: PhNCS, ethanol, reflux(waterbath), 2 h; i: NH_2_NH_2_. H_2_O, ethanol, reflux, 8 h.

Synthesis pathways adopted for the preparing the designed compounds **1–12** are illustrated in Scheme 1. According to the published procedures [20,21], the starting aminothiophene ester **1** was prepared using the cycloketone, sulfur element, ethyl cyanoacetate, and morpholine. The 2-acetamido derivative of thiophene carboxylate **2** was synthesized by reacting the 2-aminothiophene ethyl carboxylate **1** with acetic anhydride. Reacting the acetamido derivative **2** with hydrazine hydrate to afford the target structure 3-amino-2-methyl thienopyrimidine **3** was achieved according to the reported method [21]. Refluxing an equimolar amount of chloroacetyl chloride and 3-aminothienopyrimidine **3** in chloroform yielded the 2-chloro acetamido thieno[2,3-*d*]pyrimidine derivative **8** as reported [22]. The 3-methyl-4-oxothienopyrimidine pyrrolo2,5-dionederivative **4** was obtained upon heating compound **3** with 3-methylfuran-2,5-dione in CHCl_3_ for 11 h, (Scheme 1).

*N*-(3-*tert*-butylisoxazol-5-yl)-2-chloroacetamide **I-a** was prepared by gentle heating chloroacetyl chloride with 3-*tert*-butylisoxazol-5-amine) for 4 h followed by stirring at R.T. for 12 h, which upon heating with 3-aminothienopyrimidine **3** on a water bath for 12 h, afforded *N*-(3-*tert*-butylisoxazol-5-yl)acetamide **7a**. Synthesis of *N*-(5-*tert*-butylisoxazol-3-yl)-2-chloroacetamide **I-b** was performed by gently heating chloroacetyl chloride with 5-*tert*-butylisoxazol-3-amine for 4 h followed by stirring at R.T. for 12 h. Upon heating of **I-b** with 3-aminothienopyrimidine **3** on water bath for 12 h gave *N*-(5-*tert*-butylisoxazol-3-yl)acetamide **7b**, (Scheme 1).

Reaction under reflux of the acetamido thienopyrimidine **8** with the appropriate 1^ry^ amine 5-*tert*-butylisoxazol-3-amine and 3-*tert*-butylisoxazol-5-amine in DCM resulted compounds **9a** and **9b**, respectively. Moreover, reacting of **8** with 2^ry^ amines morpholine and *N*-methyl piperazine in DCM for 9 h afforded **10a** and **10b,** respectively. The 3-phenylthioureido thiophene derivative **11** was accomplished by reaction of aminothiophene **1** and phenylisothiocyanate in ethanol. Condensation of the corresponding thioureido derivative **11** with hydrazine hydrate in ethanol yielded the 3-amino-2-phenylaminothienopyrimidinone derivative **12**, (Scheme 1).

### 2.2. Biological Activity

#### 2.2.1. Antiproliferative/Cytotoxicity Assessment

**Figure 3:**
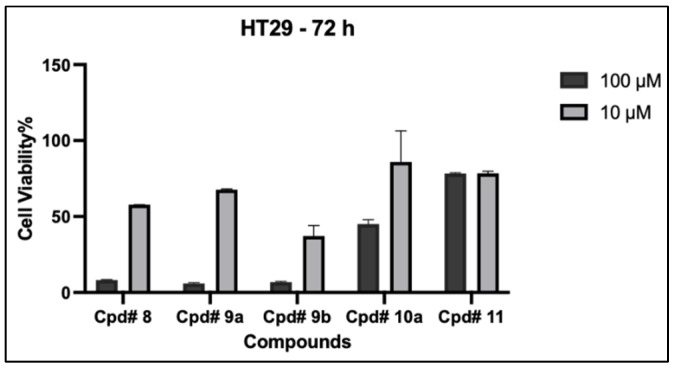
Top screening scoring synthesized compounds on the viability of HT-29. Cells were treated with 10 and 100 µM for 72 h and cell viability was determined using MTT assay. Data are expressed as mean ± SD; *n* = 3.

All synthesized compounds were subjected to preliminary antiproliferative assessment that was performed on HT-29 cells using concentrations 10 and 100 μM of synthesized compounds for 72 h and the percentage of cell viability was then calculated. The preliminary antiproliferative assay results revealed that five compounds **8**, **9a**, **9b**, **10a**, and **11** exhibited moderate to significant growth inhibitory activities against HT-29 cells. Compounds **9b** and **8** showed superior inhibitory activities at concentration 10 μM (37.18 ± 6.9 and 57.7 ± 0.76, respectively) followed by compounds **9a**, **10a**, and **11** that also showed favorable inhibitory effects at the tested doses (100 and 10 μM), Figure 3. Thus, all five compounds were further studied by a detailed dose–response relationship against three different cancerous cell lines HT-29, Hep-G2 and MCF-7.

#### 2.2.2. Detailed Dose–Response Relationship against Cancer Cells

The most promising compounds **8**, **9a**, **9b**, **10a**, and **11** were assessed to confirm their anticancer activities and calculate their IC_50_ values against three cell lines (HT-29, HepG-2 and MCF-7). Doxorubicin was used as a positive reference for the comparison of anticancer activities. The results are tabulated as IC_50_ value in the μM range, Table 1. The IC_50_ values indicated that the selected compounds showed versatile anticancer activities against the tested cell lines. Compound **9a** showed the most potent cytotoxic effect against HepG-2, and MCF-7 cell lines with IC_50s_ 6.62 ± 0.7 and 7.2 ± 1.9 µM, respectively, and more potent than doxorubicin whose IC_50s_ were 13.915 ± 2.2 and 8.43 ± 0.5 µM, respectively. The compound **9a** cytotoxicity against HT-29 was the second with IC_50_ 1.21 ± 0.34 µM after its cogner **9b** that recorded the best IC_50_ value of 0.85 ± 0.16 µM against colon cancer cell line HT-29.

Similarly, compounds **8**, **9b** and **11** showed excellent anticancer activities on the tested cell lines with IC_50_ values comparable to that of the reference compound (doxorubicin). Notably, compound **10a** exhibited weak inhibitory activity against all tested cell lines, Table 1.

Correlating the designed structure of the prepared compounds to their biological activity, introduction of the previously cited methyl pyrrole-2,5-dione moiety to the prepared nucleus in compound **4** led to weak growth inhibition at concentration 100 µM. Incorporating the primary amine “3-*tert*-butyl isoxazal-5-yl amine” which is presented in quizartinib kinase inhibitor in compound **9a** afforded significant anticancer activity in all tested cell lines HT-29, HepG-2 and MCF-7 with IC_50_ values 1.21 ± 0.34, 6.62 ± 0.7, and 7.2 ± 1.9, respectively. In a step for comparing the kinase inhibition results attributed to the isosteric replacement of 3-*tert*-butyl isoxazal-5-yl amine group by 5-*tert*-butyl isoxazal-3-yl in compound **9b**, it exhibited inhibitory activity comparable to that of **9a**. Replacing of the incorporated primary amine moieties with secondary amines “morpholine and *N*-methyl piperazine” was performed, aiming to test the effect of replacement on biological activity in compounds **10a** and **10b**, and this replacement led to a weak cytotoxic profile. Moreover, referring to sorafenib as a reported multityrosine kinase inhibitor bearing a diaryl urea moiety, we incorporated thiourea as a bio-isosteric replacement of urea at position 2 of the thiophene core, which is also a bio-isostere of the benzene ring presented in sorafenib, and structure **11** exhibited excellent inhibitory activity comparable to the reference standard against the three tested cell lines, in particular against MCF-7 as it showed an antiproliferative effect with IC_50_ 7.537 ± 0.07 μM better than the tested reference that recorded 8.43 ± 0.5 μM, Table 1.

#### 2.2.3. Selectivity Assay

In an attempt to evaluate the anticancer activity of the compounds, selectivity assay was performed and compounds’ selectivity to malignant cells was determined, reflected by their selectivity index. The selectivity index (SI) was calculated by the ratio of the IC_50_ value of the selected compounds on normal colon epithelial cells (CCD 841 CoN) to the IC_50_ value of the compounds on cancer cells (HT-29). Notably, compounds with an SI greater than 3 were proposed to have potential selectivity towards cancer cells [23,24]. As indicated in Table 2, all compounds **8**, **9a**, **9b**, and **11** demonstrated an excellent potential selectivity with SI values of 75.5, 9.4, 20.2 and 99.7, respectively. However, based on the designed strategy, this high selectivity towards cancer cells may be attributed to their ability to target oncogenic kinases.

#### 2.2.4. Target Prediction

**Figure 4:**
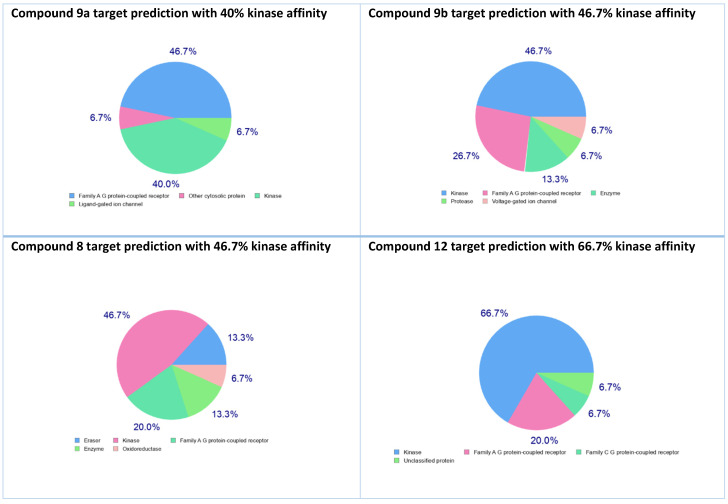
Compounds **9a**, **9b, 8**, and **12** with the highest predicted kinase affinity of 40%, 46.7%, 46.7% and 66.7% respectively.

In an attempt for further exploration of the anticancer target of the prepared compounds, in-silico target prediction was run as reported [25] on the prepared compounds, where kinase affinity was expressed in promising values. Affinity percentages range from 66.7% to 6.7%. The 2-phenylaminothienopyrimidine derivative **12** recorded the highest kinase affinity value with 66.7%, followed by the acetamido thienopyrimidine derivative **8** and 5-*Tert*-butylisoxazolylamino thienopyrimidine **9b** which expressed 46.7% kinase affinity, then the 3-*Tert*-butylisoxazolylamino analogue **9a** that recorded kinase affinity equivalent to 40%, and the rest of the compounds recorded from 40% to 6.7%, the least value 6.7% was for compound **10b**, Figure 4.

#### 2.2.5. Kinase Inhibition Activity

**Figure 5:**
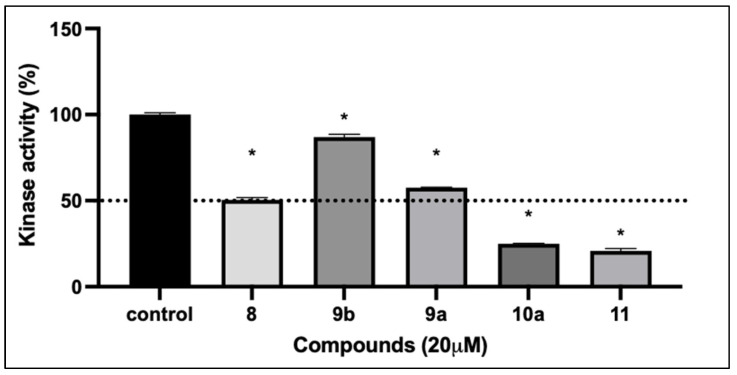
The effect of test compounds at 20 μM concentration against the activity of total intracellular kinase enzymes. Data are expressed as mean ± SD; *n* = 3. (*): Significantly different from control group (*p* value < 0.0001).

In reference to target prediction results, selected compounds were screened for their kinase inhibition activities at a single dose (20 μM). As shown in Figure 5, the acetamido derivative **8**, the 2-morphilino acetamide **10a**, and the phenylthioureidothiophene-3-carboxylate **11** exhibited potent inhibition against kinases with 49.6%, 75.1% and 79.2% inhibition, respectively. Among all derivatives, the 5-*tert*-butylisoxazolylamino thienopyrimidine **9b** expressed moderate percentage inhibition at 13.05%. Although the cytotoxic effect of compound **12** against the three selected cell lines was not that promising, the target prediction score prompted our interest to test the effect of rigidification on kinase inhibition performance in the 2-phenylaminothienopyrimidine derivative **12**, which was notably promising, as it recorded 65.3% kinase inhibition. Interestingly, a significant kinase inhibitory effect was recorded by compound **10a** with 75.1% inhibition despite its weak antiproliferative effect which can be attributed to difficulty diffusing through the cell membrane and/or inefficient cellular uptake. However, observed agreement between kinase inhibition and the selectivity index was clear with compound **11** that exhibited the highest selectivity to cancer cells with a significant kinase inhibition, Figure 5.

#### 2.2.6. Molecular Docking

**Figure 6:**
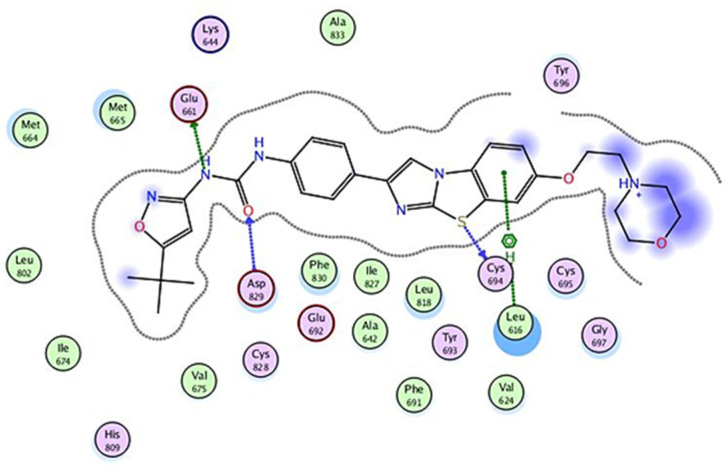
P30-re-docked ligand 2D interactions inside the active site of FLT3 receptor.

In the target prediction step, the FLT3 kinase enzyme was the kinase enzyme of high probability, and accordingly, molecular docking studies were performed on the prepared compounds that showed the most promising biological results in kinase screening to investigate their affinity for the FLT3 kinase enzyme. The target enzyme crystal structure (FLT3 kinase) bound to its co-crystallized ligand (P30-Quizartinib) was downloaded from Protein Data Bank (PDB:4XUF) [26]. An in-silico screening study of the prepared structures was carried out at the FLT3 kinase active site in comparison with the co-crystalized ligand. A redocking step was performed for validation of results, in which the co-crystalized ligand was re-docked in the ligand site, recording a binding affinity of −9.989 and RMSD deviation of 1.681 A, Figure 6. Docking results of the synthesized compounds including binding affinity score and root mean square deviation RMSD in addition to the ligand interactions with the active site residues, either hydrogen bonding or hydrophobic interactions, are tabulated below, Table 3. Receptor residues Glu 661, Cys 694, Leu 616, and Asp 829 were involved in the interaction between the re-docked ligand at the receptor pocket. Three of which, “Glu 661, Leu 616, and Asp 829” were mainly involved in most interactions between the synthesized ligands and kinase active site. Table 3.

#### 2.2.7. In-Vitro Enzyme Inhibition Assay

**Figure 7:**
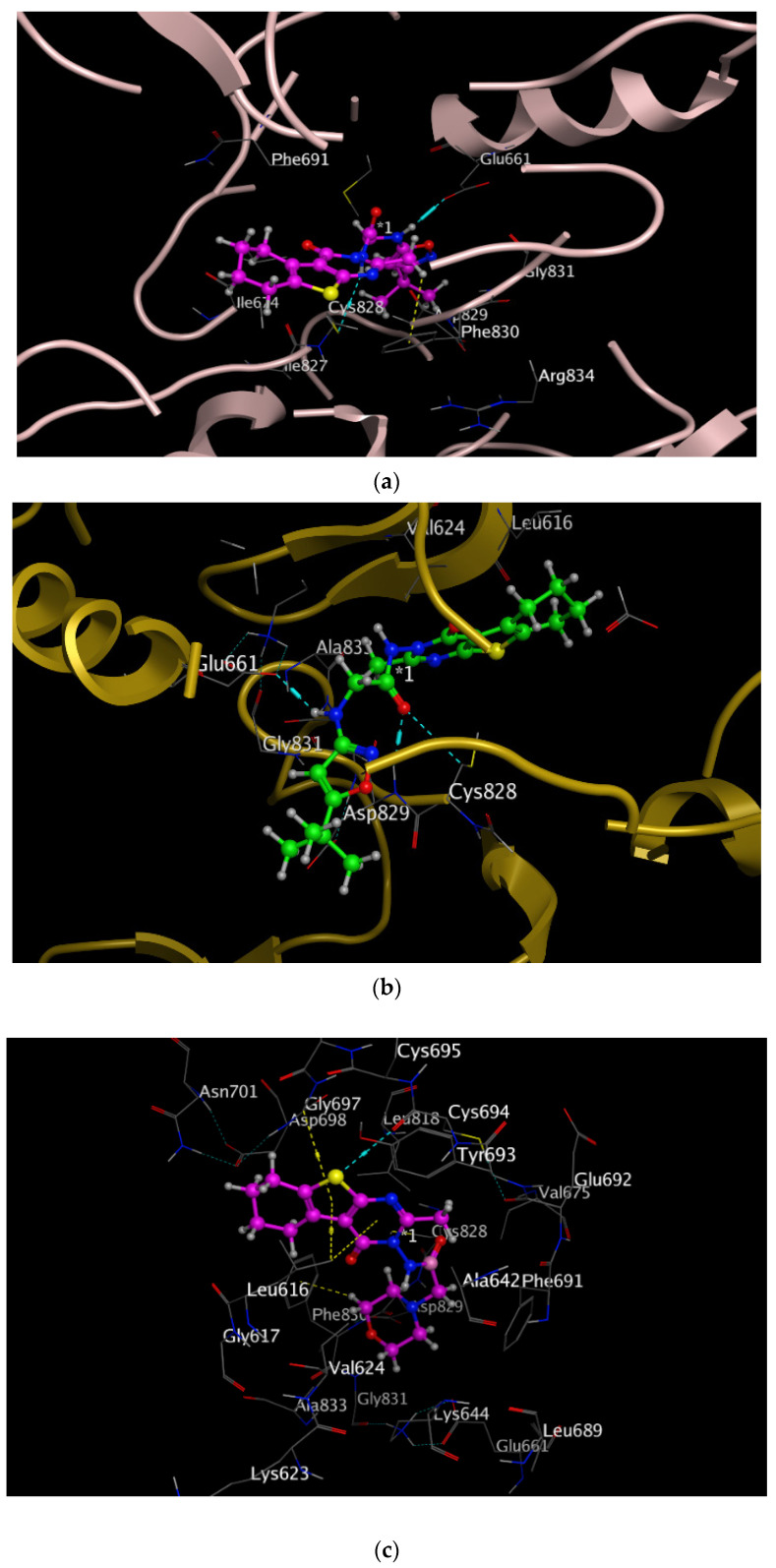
Compounds **9a**, **9b** and **10a** interactions inside the active site of FLT3 with H-bonds in cyan and H-*Pi* interaction in yellow. (**a**) **9a** (in magenta ball and stick) (**b**) **9b** (in green ball and stick). (**c**) **10a** (in magenta ball and stick).

Based on the promising biological effect of compounds **9a**, **9b**, **10a**, **11** and **12** besides their promising binding affinity in molecular docking results that are consistent with their kinase inhibitory profile, further exploration of their inhibitory activities against FLT3 kinase was performed using an *in-vitro* enzyme inhibition assay. Table 4 summarizes the inhibitory assessment of the selected compounds reflected by their IC_50_ values. The results showed that the 2-morpholinoacetamido derivative **10a**, Figure 7, which recorded receptor binding affinity -6.831 and three interactions with the receptor active site with Leu 616, Cys 694, Asp 698 amino acids, either H-bonding or H-*pi* interactions, exhibited superior FLT3 inhibitory activity among the tested compounds with IC_50_ 17.83 ± 3.8 µM followed by the tertiarybutylisoxazol-5-ylamino acetamide derivative **9a**, Figure 7, whose binding affinity was -7.785 and showed H and H-*pi* interactions with the receptor pocket at Glu 661, Cys 828, then the phenylamino-thienopyrimidinone derivative **12** which displayed receptor binding energy of −6.632 and receptor interactions at Glu 692, Asp 829, Phe 691 amino acids. Compounds **9a** and **12** IC_50s_ were 20.4 ± 2.8 and 27.22 ± 5.6 µM, respectively. Despite their excellent antiproliferative activities against cancer cells and their promising binding affinity in modeling studies, compounds **9b** and **11** exhibited moderate FLT3 enzyme inhibitory activities, recording IC_50_ values 47.64 ± 9.3 and 40.05 ± 5.5 µM, respectively, Table 4.

#### 2.2.8. Pharmacokinetics Evaluation and Drug Likeness

Bioinformatics assessment was performed on compounds that scored the best biological activities values, i.e., **8**, **9a**, **9b**, **10a**, **11** and **12,** using both Molsoft and Swiss ADME [25]. Regarding drug-likeness scores, compounds are considered good drug-like candidates when they express positive value. As illustrated in the table below, Table 5, all the tested compounds expressed positive values ranging from 0.52 to 1.06, among which compound **10a** that recorded the best drug-like score of 1.06. In reference to Lipinski’s rule of five [27], all the investigated compounds are considered promising “drug-like” molecules with good bioavailability, as they all had no Lipinski’s rule violations. All the tested compounds recorded log P < 5, which indicated their strong tolerance by cell membranes. In addition, molecular weights are ≤500 and the topological polar surface area (TPSA) values range from 92 to 130. The number of hydrogen bond acceptors range from 2 to 5 acceptors while the number of hydrogen bond donors are either 1 or 2. As tabulated below, the blood brain barrier (BBB) score is between 0 to 6 as referenced [28], recording a minimum value of 3.09 and maximum of 4.29, Table 5.

## 3. Materials and Methods

### 3.1. Chemistry

Melting points were measured and values were uncorrected. The reactions’ duration was determined using thin layer chromatography (TLC) technique, which was performed using plates of aluminum oxide sheets coated with silica gel. A Bruker advance 400 MHz NMR spectrometer was used for ^1^H NMR. A Bruker advance 100 MHz spectrometer(microanalytical lab, Cairo University, Cairo, Egypt) applied for measurement ^13^C NMR spectral analysis.

The starting key structures thiophene 2-aminocarboxylate **1**, acetylamino benzothiophene carboxylate cogner **2**, and 2-methyl-3-amino thienopyrimidine derivative **3** were synthesized as previously reported [29,30].

*3-Methyl-1-(2-methyl-4-oxo-5,6,7,8-tetrahydrobenzo[4,5]thieno[2,3-d]pyrimidin-3(4H)-yl)-1H-pyrrole-2,5-dione:* **4**

Reaction of 10 mmol of **4** with 15 mmol of 3-methylfuran-2,5-dione in 10 mL CHCl_3_ for 11 h to give yellow powder of pyrrolodione derivative. Yield: 84%, m.p. 125–127 °C; ^1^H-NMR (400 MHz, DMSO-d6) ppm: 1.24 (s, 3H, CH_3_ at C2-pyrimidine), δ 1.72–2.98 (m, 8H, cyclohexane), 2.33 (s, 3H, CH_3_ at C2-pyrrol), 5.98 (s, 1H, pyrrol ring); ^13^C-NMR (100 MHz, DMSO-d6) ppm: δ 16.3, 18.9, 20.4, 23.5, 23.8, 25.3, 117.4, 125.3, 134.6,138.8, 144.4,158.3, 163.2, 165.2; analysis for: C_16_H_15_N_3_O_3_S [329]: CHN calcd. C, 58.34; H, 4.59; N, 12.76; found: C, 58.20; H, 5.04; N, 12.88.

*1-(2-Methyl-4-oxo-5,6,7,8-tetrahydrobenzo[4,5]thieno[2,3-d]pyrimidin-3(4H)-yl)-3-phenylthiourea:* **5**

Phenyl isothiocyanate (40 mmol) was added to the 3-aminothienopyrimidine **3** (10 mmol) in dichloromethane (30 mL), the mixture was refluxed for 14 h. Excess solvent was removed under vacuum, the remained solid was collected, dried, and crystallized from absolute ethanol. Yield 73%, m.p: 138–140 °C; ^1^H-NMR (400 MHz, DMSO-d6) ppm: 1.20–3.00 (m, 3H, CH_3_ at C2-pyrimidine and 8H, cyclohexane), 5.50 (s, 1H, exchangeable NH), 6.90–7.12 (m, aromatic-5H), 12.00 (s, 1H, D_2_O exchangeable NH). ^13^C-NMR (DMSO, d6) ppm δ: 20.2, 21.5, 23.0, 23.2, 25.8, 119.7, 124.3, 125.1, 126.5, 126.5, 129.2, 129.8, 139.2, 141.4, 155.6, 162.1, 164.8, 179.7. Analysis for: C_18_H_18_N_4_OS_2_ [370]: CHN calcd. C, 58.35; H, 4.90; N, 15.12; found: C, 58.66; H, 5.04; N, 15.33.

*2-Methyl-3-(5-oxo-3-phenyl-2-thioxoimidazolidin-1-yl)-5,6,7,8-tetrahydrobenzo*[4,5]*thieno[2,3-d]pyrimidin-4(3H)-one:***6**

Chloroacetyl chloride (1.12 mL, 10 mmol) was added drop wise to an equimolar amount of phenylthioureido-thienopyrimidine derivative (10 mmol) **5** in dry benzene (15 mL) and 4 dps of TEA, refluxed for 11 h, left stirring overnight, then poured gently on ice/water, the separated precipitate was then collected, dried, and crystallized from diethyl ether. Yield: 69%, m.p. 198–200 °C; ^1^H-NMR (400 MHz, DMSO-d6) ppm: 1.24–2.98 (m, 3H, CH_3_ at C2-pyrimidine and 8H, cyclohexane), 4.98 (s, 2H, CH_2_-thioxoimidazole), 7.21–7.45 (m, aromatic-5H). ^13^C-NMR (DMSO, d6) ppm δ: 20.4, 21.6, 22.8, 22.8, 25.8, 61.3, 124.3, 125.7, 126.0, 126.0, 131.2, 131.4, 139.9, 141.3, 147.3, 156.0, 162.1, 164.4, 168.9, 177.6. Analysis for: C_20_H_18_N_4_O_2_S_2_ [410]: CHN calcd. C, 58.52; H, 4.42; N, 13.65; found: C, 58.58; H, 4.44; N, 13.69.

General procedures for **7a** and **7b**:

Preparation of *N*-(3-*tert*-butylisoxazol-5-yl)-2-chloroacetamide **I-a** and *N*-(5-*tert*-butylisoxazol-3-yl)-2-chloroacetamide **I-b:** Chloroacetyl chloride (1.12 mL, 10 mmol) was added drop wise to the appropriate 1^ry^ amine namely 3-*tert*-butylisoxazol-5-amine and 5-*tert*-butylisoxazol-3-amine, respectively, in 20 mL chloroform and 4 dps of TEA. The reaction was gently refluxed on water bath 80 °C for 4 h, then stirring was continued overnight at room temperature. The reaction mixture was then poured in a dropwise manner onto ice/water. The separated solid was filtered, left to dry then recrystallized from absolute ethanol.

A reaction mixture of 3-aminothienopyrimidine **3** and the proper chloroacetamide namely, *N*-(3-*tert*-butylisoxazol-5-yl)-2-chloroacetamide **I-a** and *N*-(5-*tert*-butylisoxazol-3-yl)-2-chloroacetamide **I-b was** heated gently at 80 °C on a water bath using 4 dps of TEA for 12 h to yield *N*-(3-*tert*-butylisoxazol-5-yl)acetamide **7a** and *N*-(5-*tert*-butylisoxazol-3-yl)acetamide **7b,** respectively.

*2-(2-Methyl-4-oxo-5,6,7,8-tetrahydrobenzo[4,5]thieno[2,3-d]pyrimidin-3(4H)-ylamino)-N-(3-tert-butylisoxazol-5-yl)acetamide:* **7a**

Yield: 73%, m.p. 120–122 °C; ^1^H-NMR (400 MHz, DMSO-d6) ppm: δ 1.25 (s, 3H, CH_3_ at C2-pyrimidine), 1.78–3.00 (m, 8H, cyclohexane), 1.33 (s, 9H *tert*-butyl), 3.50 (s, 1H isoxazole), 3.65 (s, 2H, CH_2_), 8.00 (s, 1H exchangeable NH), 12.50 (s, 1H exchangeable NH).^13^C-NMR (DMSO, d6) ppm δ: (19.3, 20.4, 22.6, 23.5, 25.4, 30.8, 33.6, 33.6, 33.6, 58.4, 99.0, 125.0, 139.6, 150.1, 158.8, 155.4, 163.1, 164.4, 168.5, 174.8); analysis for: C_20_H_25_N_5_O_3_S [415]: CHN calcd. C, 57.81; H, 6.06; N, 16.85; found: C, 57.99; H, 6.08; N, 16.91.

*2-(2-Methyl-4-oxo-5,6,7,8-tetrahydrobenzo[4,5]thieno[2,3-d]pyrimidin-3(4H)-ylamino)-N-(5-tert-butylisoxazol-3-yl)acetamide:* **7b**

Yield: 64%, m.p. 128–130 °C; ^1^H-NMR (400 MHz, DMSO-d6) ppm: δ 1.22 (s, 3H, CH_3_ at C2-pyrimidine), 1.31 (s, 9H *tert*-butyl), 1.72–2.98 (m, 8H, cyclohexane), 3.21 (s, 1H isoxazole), 3.55 (s, 2H, CH_2_), 12.00 (s, 1H exchangeable NH), 13.00 (s, 1H exchangeable NH). ^13^C-NMR (DMSO, d6) ppm δ: 17.9, 22.4, 22.4, 25.3, 25.4, 29.8, 31.6, 33.4, 33.4, 62.6, 119.0, 126.2, 139.9, 152.6, 156.7, 159.1, 163.8, 166.9, 171.5, 174.8); analysis for: C_20_H_25_N_5_O_3_S [415]: CHN calcd. C, 57.81; H, 6.06; N, 16.85; found: C, 58.02; H, 6.14; N, 16.88.

*2-Chloro-N-(2-methyl-4-oxo-5,6,7,8-tetrahydrobenzo[4,5]thieno[2,3-d]pyrimidin-3(4H)-yl)acetamide:* **8**

An equimolar amount (10 mmol) of Chloroacetyl chloride and 3-aminothienopyrimidine **3** in chloroform (20 mL), refluxed gently 110 °C for 4h then stirring was continued overnight at room temperature. The reaction mixture was then poured onto ice/water. The separated solid was filtered, left to dry then recrystallized from absolute ethanol into yellowish white crystals. Yield: 71%, m.p. 200–202 °C; ^1^H-NMR (DMSO) δ (ppm), 1.26–3.34 (m, 3H, C2―H, 8H of cyclohexane), 4.30 (s, 2H, COCH_2_), 11.32 (s, 1H, NH D_2_O exchangeable). ^13^C NMR (DMSO-d6) δ ppm: 18.97, 20.60, 26.50, 27.81, 28.92, 60.67,117.4, 125.89, 134.34, 138.08, 155.53, 158.90, 166.29. Analysis for: C_13_H_14_ClN_3_O_2_S [311.5]: CHN calcd. C, 50.08; H, 4.53; N, 13.48; found: C, 50.11; H, 4.55; N, 13.62.

General procedures for **9a** and **9b**: Reaction of equimolar amounts of chloroacetamido derivative **8** with the proper primary amine (3-*tert*-butylisoxazol-5-amine and 5-*tert*-butylisoxazol-3-amine) in DCM under reflux for 11 h adding 3–4 dps of TEA. The mixture was cooled and poured onto iced water, the formed solid was filtered under vacuum, then crystallized from ethanol to afford compounds **9a** and **9b,** respectively.

*2-(3-Tert-butylisoxazol-5-ylamino)-N-(2-methyl-4-oxo-5,6,7,8-tetrahydrobenzo[4,5]thieno[2,3-d]pyrimidin-3(4H)-yl)acetamide:* **9a**

Yield: 82%, m.p. 89–92 °C; ^1^H-NMR (400 MHz, DMSO-d6) ppm: δ 1.34–3.00 (m, 8H, cyclohexane), 1.24 (s, 3H, CH_3_ at C2-pyrimidine), 2.20 (s, 9H, t-butyl), 3.28 (s, 1H, CH-isoxazole), 4.89 (s, 1H, NH D_2_O exchangeable), 4.38 (s, 2H, CH_2_-NH), 12.00 (s, 1H, NH D_2_O exchangeable). ^13^C-NMR (100 MHz, DMSO-d6) ppm: δ 18.9, 20.4, 23.5, 23.8, 25.3, 35.1, 35.1, 35.1, 40.2, 57.0, 95.1, 117.4, 125.3, 138.8, 144.4, 158.3, 161.0, 163.2, 164.0, 165.2; analysis for: C_20_H_25_N_5_O_3_S [415]: CHN calcd. C, 57.81; H, 6.06; N, 16.85; found: C, 58.20; H, 6.04; N, 16.88.

*2-(5-Tert-butylisoxazol-3-ylamino)-N-(2-methyl-4-oxo-5,6,7,8-tetrahydrobenzo[4,5]thieno[2,3-d]pyrimidin-3(4H)-yl)acetamide:* **9b**

Yield: 77%, m.p. 98–100 °C; ^1^H-NMR (400 MHz, DMSO-d6) ppm: δ 1.62–2.98 (m, 8H, cyclohexane), 1.24 (s, 3H, CH_3_ at C2-pyrimidine), 2.13 (s, 9H, t-butyl), 3.28 (s, 1H, CH-isoxazole), 4.00 (s, 1H, NH D_2_O exchangeable), 4.95 (s, 2H, CH_2_-NH), 11.32 (s, 1H, NH D_2_O exchangeable). ^13^C-NMR (100 MHz, DMSO-d6) ppm: δ 20.2, 21.3, 23.1, 23.1, 24.6, 33.0, 33.0, 35.2, 41.0, 54.7, 93.3, 119.4, 124.1, 134.9, 143.7, 157.5, 159.8, 162.1, 164.3, 164.9; analysis for: C_20_H_25_N_5_O_3_S [415]: CHN calcd. C, 57.81; H, 6.06; N, 16.85; found: C, 57.98; H, 6.11; N, 16.89.

General procedures for **10a** and **10b**: Reflux of a mixture of the chloroacetamido derivative **8** with the appropriate secondary amine namely morpholine and methyl piperazine, and 3–4 dps of TEA in DCM for 9 h. The mixture was then left to cool, poured onto ice/water, filtered under vacuum and crystalized from diethyl ether to afford compounds **10a** and **10b,** respectively.

*N-(2-methyl-4-oxo-5,6,7,8-tetrahydrobenzo[4,5]thieno[2,3-d]pyrimidin-3(4H)-yl)-2-morpholinoacetamide:* **10a**

Yield: 79%, m.p. 222–225 °C; ^1^H-NMR (400 MHz, DMSO-d6) ppm: δ 1.77–2.55 (m, 8H, cyclohexane), 1.24 (s, 3H, CH_3_ at C2-pyrimidine), 2.40–3.51 (m, 8H-morpholine ring), 3.95 (s, 2H, CH_2_), 9.00 (s, 1H, NH D_2_O exchangeable). ^13^C-NMR (100 MHz, DMSO-d6) ppm: δ 19.5, 20.4, 23.5, 23.8, 25.3, 55.1, 55.4, 59.1, 66.1, 66.1, 117.7, 126.0, 139.8, 154.4, 159.8, 163.0, 169.2; analysis for: C_17_H_22_N_4_O_3_S [362]: CHN calcd. C, 56.33; H, 6.12; N, 15.46; found: C, 56.28; H, 6.10; N, 15.81.

*N-(2-methyl-4-oxo-5,6,7,8-tetrahydrobenzo[4,5]thieno[2,3-d]pyrimidin-3(4H)-yl)-2-(4-methylpiperazin-1-yl)acetamide:* **10b**

Yield: 65%, m.p. 187–189 °C; ^1^H-NMR (400 MHz, DMSO-d6) ppm: δ 1.77–2.55 (m, 8H, cyclohexane), 1.24 (s, 3H, CH_3_ at C2-pyrimidine), 2.4 (s, 3H, CH_3_ piperazino), 3.00–3.50 (m, 8H, piperazine ring), 4.00 (s, 2H, CH_2_), 9.00 (s, 1H, NH D_2_O exchangeable). ^13^C-NMR (100 MHz, DMSO-d6) ppm: δ 20.5, 22.4, 24.3, 24.5, 25.8, 56.2, 56.4, 57.1, 57.6, 59.2, 119.7, 129.0, 142.8, 146.4, 159.8, 161.2, 163.0, 179.0; analysis for: C_18_H_25_N_5_O_2_S [375]: CHN calcd. C, 57.58; H, 6.71; N, 18.65; found: C, 57.55; H, 6.70; N, 18.51.

*Ethyl-2-(3-phenylthioureido)-5,6,7,8-tetrahydrobenzo[b]thiophene-3-carboxylate:* **11**

The 2-Aminothiophene derivative **1** (2.40 g, 10 mmol) was dissolved in hot ethanol (10 mL). While stirring and in a dropwise manner, phenyl isothiocyanate (~2 mL, 15 mmol) was added. The reaction mixture was gently refluxed on water bath for 2 h then left overnight to cool at room temperature. The separated solid was filtered, washed, and yellow crystals were obtained using ethanol for crystallization. Yield of 92%, m.p. 170–172 °C; ^1^H-NMR (DMSO) δ (ppm) 1.22 (t, 3H, CH_3_ at C2-pyrimidine), 1.34–2.97 (m, 8H cyclohexane) 5.00 (q, 2H, CH_2_―CH_3_), 7.50–7.89 (m, aromatic-5H), 9.00 (s, 1H, NH, D_2_O exchangeable), 11.50 (s, 1H, NH, D_2_O exchangeable). ^13^C NMR (DMSO-d6) δ ppm: 14.4, 19.2, 25.5, 25.4, 27.7, 60.4, 113.6, 124.5, 126.3, 126.3, 128.4, 129.8, 131.6, 138.3, 141.2, 161.1, 163.4, 175.4. Analysis for: C_18_H_20_N_2_O_2_S_2_ [360]: CHN calcd. C, 59.97; H, 5.59; N, 7.77; found: C, 60,11; H, 6.04; N, 7.79.

*3-Amino-2-phenylamino-5,6,7,8-tetrahydrobenzo[4,5]thieno[2,3-d]pyrimidin-4(3H)-one:* **12**

A mixture of the thioureido derivative **11** (3.70 g, 10 mmol) and hydrazine hydrate (~1 mL, 20 mmol) was refluxed in 10 mL ethanol for 8 h, left to cool, the separated solid was filtered, dried then recrystallized from ethanol. Yellow powder was collected. Yield: 71%, m.p. 198–200 °C; ^1^H-NMR (DMSO) δ (ppm): 1.39–3.11 (m, 8H cycloheptane), 2.76 (s, 2H, NH_2_, D_2_O exchangeable), 7.50–7.99 (m, Ar―5H), 13.55 (s, 1H, NH, D_2_O exchangeable). ^13^C NMR (DMSO-d6) ppm: 20.4, 23.5, 23.5, 25.0, 114.7, 114.7, 116.8, 117.8, 122.5, 126.9, 127.5, 139.2, 145.1, 152.4, 159.2, 162.8. Analysis for: C_16_H_16_N_4_OS [312]: CHN calcd. C, 61.52; H, 5.16; N, 17.90; found: C, 61.66; H, 5.18; N, 18.01.

### 3.2. Biology

#### 3.2.1. In Vitro Anti-Proliferative Activities

Cell Culture:

Three cancer cell lines were used in the antiproliferative activities experiments, namely, breast cancer cell line (MCF-7) and human hepatoma cell line (HepG-2), and Colorectal cell line (HT-29). The source of cell lines is the health sciences research center-core lab- at princess Nou-rah bint Abdulrahman university. All cell lines were maintained in their optimum media (RPMI-1640 or DMEM) supplemented with 10% fetal bovine serum, 100 U/mL penicillin, and 100 μg/mL streptomycin and incubated in a humidified atmosphere at 37 °C with 5% CO_2_.

Cell Viability Assay

The MTT assay was used to evaluate the anti-proliferative effect of the synthesized compounds against three human cancer cell lines hepatocellular carcinoma (HepG-2), breast cancer (MCF-7) and Colorectal carcinoma (HT-29). For the preliminary screening, HT-29 cells were seeded in 96-well plates (Thermo Scientific, Waltham, MA, USA)(103 cells/well) and incubated for 24 h before the treatment. Then, cells were treated with two concentrations (10 and 100 µM) of the tested compounds for 72 h. After the exposure, MTT assay protocol was performed as described [31]. After evaluation of the preliminary anticancer activity, the promising synthesized compounds (**8**, **9a**, **9b**, **10a**, and **11**) were selected and examined against three cell lines (HepG-2, MCF-7, and HT-29). Cells were exposed to serial concentration (0.1–100 μL) of the selected compounds and dose–response curves were determined to calculate their IC_50_ values.

Selectivity Index (SI)

The selectivity degree of the most potent synthesized compounds was examined in non-cancerous intestinal epithelial cell line (CCD 841 CoN). Cells were plated in 96-well plates (103 cells/well), and after 24 h, the compounds in concentrations (0.1 to 100 μM) were incubated for 72 h. MTT procedure was then performed, the ratio of IC_50_ values of the compounds on non-cancerous cells (CCD 841 CoN) to the IC_50_ values of the compounds on cancer cells (HT-29) was determined as reported, [23,24] using the following equation, SI = IC_50_ of normal cells/IC_50_ of cancer cells.

Kinase inhibition assessment

Inhibitory activity against kinases of the selected compounds was examined using homogeneous protein kinases extracted from HT-29 cell line. The screening assay was performed in a single dose concentration (20 μM) by using ADP-Glo assay (Promega, Madison, WI, USA).

Briefly, protein kinases were added into a 96-well plate followed by addition of the synthesized compounds at the tested dose (20 μM). After 45 min of exposure, kinase activity was assessed using a luminescent luciferase/luciferin reaction. Luminescence quantification was measured by using a Varioskan™ LUX multimode microplate reader (Thermo Scientific, Waltham, MA, USA). Kinase inhibition effect of tested compounds was expressed as the percentage of remaining kinase activity relative to vehicle kinase reaction.

#### 3.2.2. In-Vitro Enzyme Inhibition Assay

FLT3 Kinase Assay Kit (BD Biosciences, San Diego, CA, USA) was used to determine the inhibitory selectivity of the selected compounds towards the FLT3 enzyme. briefly, serial dilution of the selected compounds was incubated with FLT3 (1.5 ng/µL) for 45 min in a kinase reaction buffer. The FLT3 enzyme reaction was halted and measured by quantifying the amount of ADP produced during the enzyme reaction. Luminescence values were measured by a Varioskan™ LUX multimode microplate reader (Thermo Scientific, Waltham, MA, USA). IC_50_ was calculated with nonlinear regression compared with untreated reaction using Prism version 8.4.3 (GraphPad).

### 3.3. Molecular Modeling Study

*Protein preparation*: targeted enzyme was downloaded from Protein Data Bank along with its bounded co-crystallized ligand (PDB:4XUF). Preparation of the downloaded protein was performed by optimization of receptor amino acids residues, deleting water molecules, 3D-protonation and correcting of connections errors.

*Compounds optimization*: all compounds were drawn and imported from chem-draw, structure optimization was done, as well as 3D protonation. All bonded Van der Waals, electrostatics, and restraints were enabled. Compounds were saved after calculating partial charges and energy minimization in mol2 format.

*Applied protocol for docking using MOE* [32]: induced fit was the applied protocol for docking. Active site was defined by ligand atoms, alpha spheres were used as placement guide. MDB file was chosen as ligand and rotatable bonds were allowed. The docking placement methodology as well as initial scoring and post placement refinement were set as per the program defaults. Gradient was set at 0.05 for minimizing energy, and MMFF94X was set as the force field.

## 4. Conclusions

In the present study, we were interested in evaluating the anticancer and kinase inhibition effect of newly synthesized thieno[2,3-*d*]pyrimidine derivatives targeting the FLT3 kinase enzyme. In reference to reported active compounds, a strategy was designed for synthesis of a thienopyrimidine targeting kinase enzyme. Synthesized compounds were tested for their cytotoxic activity, where compounds **9a** and **9b** showed the most potent cytotoxic effect against HepG-2 cell line with an IC_50s_ 6.62 ± 0.7 and 9.11 ± 0.3 µM, respectively, superior to the reference standard that demonstrated an IC_50_ of 13.915 ± 2.2 µM. Compounds **9b**, **9a** and **11** recorded the best IC_50_ values (0.85 ± 0.16, 1.21 ± 0.34, and 1.78 ± 0.6) µM, respectively, against colon cancer cell line HT-29, which is better than that of doxorubicin that demonstrated an IC50 of 1.4 ± 1.16 µM. Regarding MCF-7, compounds **9a** and **11** expressed better antiproliferative activity than doxorubicin reflected by their IC_50__s_ of 7.2 ± 1.9 and 7.53 ± 0.07 µM compared to an IC_50_ of 8.43 ± 0.5 µM recorded by the standard reference. In an attempt to evaluate compounds’ selectivity to malignant cells, selectivity assay was performed. Interestingly, all the tested compounds demonstrated excellent potential selectivity reflected by their selectivity index (SI) ranges (20.2 to 99.7), where compound **11** showed the most marked cancer cells selectivity with an SI of 99.7. Exploration of the anticancer target was done using in-silico target prediction where kinase affinity was expressed in promising values (≥40%), FLT3 kinase enzyme was the kinase enzyme of highest probability. Accordingly, molecular docking studies were performed on the prepared compounds which showed promising binding affinity for the FLT3 kinase enzyme and the main interactions between the synthesized ligands and kinase active site were similar to those between the co-crystallized ligand and the receptor. The binding affinity range from -8.479 to -6.632 and the RMSD values range from 1.015 to 1.778 A. Further biological exploration was performed using an in-vitro FLT3 kinase enzyme inhibition assay. The results showed that the 2-morpholinoacetamido derivative **10a** exhibited superior FLT3 inhibitory activity among the tested compounds with IC_50_ 17.83 ± 3.8 µM followed by the tertiarybutylisoxazol-5-ylamino acetamide derivative **9a** whose IC_50_ value was 20.4 ± 2.8 µM then the phenylaminothienopyrimidin-one derivative **12** with IC_50_ 27.22 ± 5.6 µM. Pharmacokinetics assessment was performed on compounds that scored the best biological activities values. Notably, all the investigated compounds were considered as “drug-like” molecules, best of which was compound **10a** with drug likeness score of 1.06. The tested compounds expressed promising bioavailability and had no Lipinski’s rule violations.

## Figures and Tables

**Table 1:** Results of anti-proliferative activities of the tested compounds on three cancer cell lines (IC_50_ (μM)).

Compound No.	Anti-Proliferative Activities in Different Cancer Cell Lines (IC_50_ (μM))		
	HT-29	HepG-2	MCF-7
**8**	2.0 ± 0.62	13.47 ± 2.7	11.0 ± 0.8
**9a**	1.21 ± 0.34	6.62 ± 0.7	7.2 ± 1.9
**9b**	0.85 ± 0.16	9.11 ± 0.3	16.26 ± 2.3
**10a**	167.4 ± 8.15	187.55 ± 28.2	104.83 ± 36.4
**11**	1.78 ± 0.6	16.38 ± 5.7	7.537 ± 0.07
**Dox**	1.4 ± 1.16	13.915 ± 2.2	8.43 ± 0.5

**Table 2:** The selectivity index (SI) which represents IC_50_ for normal cell line (CCD 841)/IC_50_ for cancerous cell line (HT29) after 72 h of exposure to the tested compounds.

Compound No.	IC_50_ of Non-Cancer Cells CCD 841 (μM)	Selectivity Index (SI) (IC_50_ of CCD 841/IC_50_ of HT29 (μM))
**8**	151.0 ± 20.2	75.5
**9a**	11.3 ± 5.6	9.4
**9b**	17.2 ± 1.4	20.2
**11**	177.5 ± 20.4	99.7

**Table 3:** Receptor binding affinity, RMSD values and residues involved in the interaction at the FLT3 active site.

Compound No.	Binding Affinity	Root Mean Square Deviation (RMSD)	Amino Acids Involved in Interactions at the Active Site
**9a**	−7.785	1.754	Glu 661, Cys 828.
**9b**	−8.479	1.015	Glu 661, Cys 828, Asp 829.
**10a**	−6.831	1.760	Leu 616, Cys 694, Asp 698.
**11**	−7.671	1.467	Glu 692, Cys 694, Asp 829, Phe 691
**12**	−6.632	1.778	Glu 692, Asp 829, Phe 691
**P30**	−9.989	1.681	Glu 661, Cys 694, Asp 829, Leu 616.

**Table 4:** IC_50_ values in µM reflecting FLT3 kinase inhibitory activity of the selected compounds.

Compound No.	IC_50_ (µM) *
**9a**	20.4 ± 2.8
**9b**	47.64 ± 9.3
**10a**	17.83 ± 3.8
**11**	40.05 ± 5.5
**12**	27.22 ± 5.6

* Data are expressed as mean ± SD; *n* = 3.

**Table 5:** Results of pharmacokinetics evaluation for the most promising kinase inhibitors.

Molecule	MW	#H-Bond Acceptors	#H-Bond donors	MR (Molar Refractivity)	TPSA	iLog*P*	BBB	Bioavailability	Drug Likeness
**8**	311.79	3	1	81.12	92.23	1.85	4.17	0.55	0.77
**9a**	415.51	5	2	114.49	130.29	3.11	3.09	0.55	0.90
**9b**	415.51	5	2	114.49	130.29	2.88	3.10	0.55	0.90
**10a**	362.45	5	1	101.34	104.7	2.45	3.60	0.55	1.06
**11**	360.49	2	2	103.76	110.69	3.46	4.29	0.55	0.52
**12**	312.39	2	2	91	101.18	2.63	4.01	0.55	0.57

# means (number).

## Data Availability

Data is contained within the article and supplementary material.

## Supplementary Materials

The following are available online at https://www.mdpi.com/article/10.3390/ph15020170/s1.
