# Supplementary material for: In-Silico Screening of Novel Synthesized Thienopyrimidines Targeting Fms Related Receptor Tyrosine Kinase-3 and Their In-Vitro Biological Evaluation
Source: Pharmaceuticals (Basel). 2022 Jan 29;15(2):170. doi: 10.3390/ph15020170 (PMC8880588; doi:10.3390/ph15020170)
Supplement: Supplementary file 1 [file pharmaceuticals-15-00170-s001.zip › pharmaceuticals-1570041-supplementary.pdf]

In-Silico Screening of Novel Synthesized Thienopyrimidines Targeting Fms Related Receptor Tyrosine Kinase-3 and Their *In-Vitro* Biological Evaluation

Elshaymaa I. Elmongy 1,2,*, Najla Altwaijry 1, Nashwah G. M. Attallah 1,3, Manal Mubarak AlKahtani 4 and Hanan Ali Henidi 4

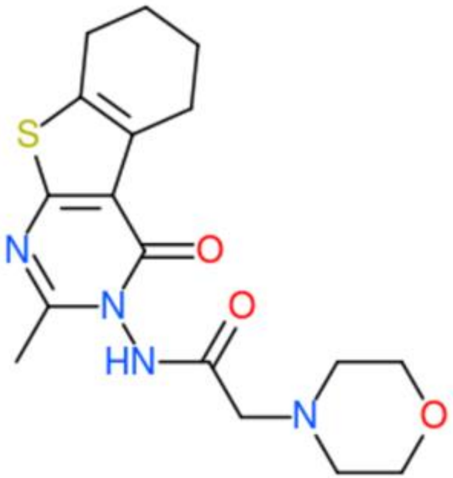

Molecular Properties and Drug-likeness.

Molecular formula: C17 H22 N4 O3 S
Molecular weight: 362.14
Number of HBA: 6
Number of HBD: 1
MolLogP : 0.94
MolLogS : -1.40 (in Log(moles/L)) 14550.50 (in mg/L)
MolPSA : 62.31 A^2
MolVol : 385.55 A^3
pKa of most Basic/Acidic group : 4.76 / 10.80

$$pK_\mathrm{a} = -\log_{10} K_a = \log_{10} \frac{[\mathrm{HA}]}{[\mathrm{A}^-][\mathrm{H}^+]}$$

BBB Score : 3.60 The Blood-Brain Barrier (BBB) Score: 6-High,0-Low *(DOI: 10.1021/acs.jmedchem.9b01220)*
Number of stereo centers: 0

Molecular Properties and Drug-likeness.

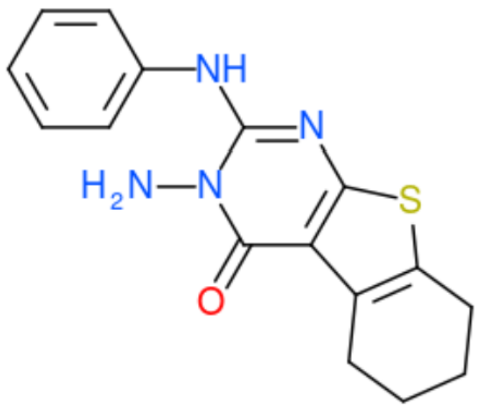

Molecular formula: C16 H16 N4 O S
Molecular weight: 312.10
Number of HBA: 4
Number of HBD: 3
MolLogP : 3.14
MolLogS : -3.71 (in Log(moles/L)) 60.86 (in mg/L)
MolPSA : 50.87 A^2
MolVol : 307.14 A^3
pKa of most Basic/Acidic group : -2.07 / 15.42

$$pK_{\mathrm{a}} = -\log_{10} K_a = \log_{10} \frac{[\mathrm{HA}]}{[\mathrm{A}^-][\mathrm{H}^+]}$$

BBB Score : 4.01 The Blood-Brain Barrier (BBB) Score: 6-High,0-Low *(DOI: 10.1021/acs.jmedchem.9b01220)*
Number of stereo centers: 0

Molecular Properties and Drug-likeness.

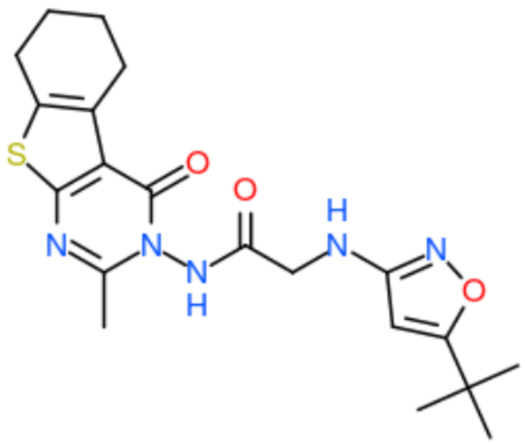

Molecular formula: C20 H25 N5 O3 S
Molecular weight: 415.17
Number of HBA: 6
Number of HBD: 2
MolLogP : 3.23
MolLogS : -3.75 (in Log(moles/L)) 73.61 (in mg/L)
MolPSA : 83.28 A^2
MolVol : 443.53 A^3
pKa of most Basic/Acidic group : 3.09 / 11.69

$$pK_\mathrm{a} = -\log_{10} K_a = \log_{10} \frac{[\mathrm{HA}]}{[\mathrm{A}^-][\mathrm{H}^+]}$$

BBB Score : 3.10 The Blood-Brain Barrier (BBB) Score: 6-High,0-Low *(DOI: 10.1021/acs.jmedchem.9b01220)*
Number of stereo centers: 0

H-NMR

4

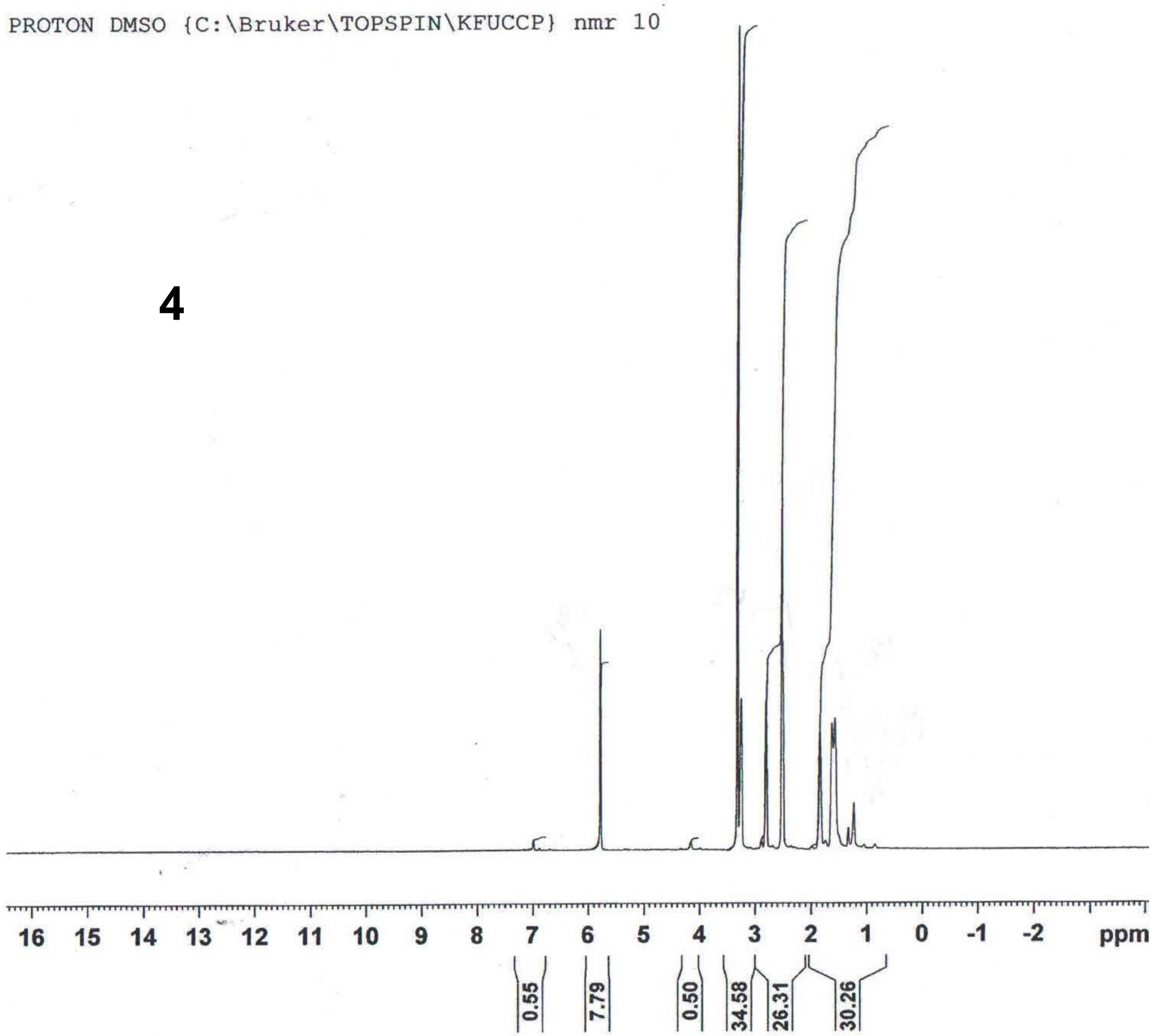

PROTON DMSO {C:\Bruker\TOPSPIN\KFUCCP} nmr 10
16 15 14 13 12 11 10 9 8 7 6 5 4 3 2 1 0 -1 -2 ppm
0.55
7.79
0.50
34.58
26.31
30.26

5

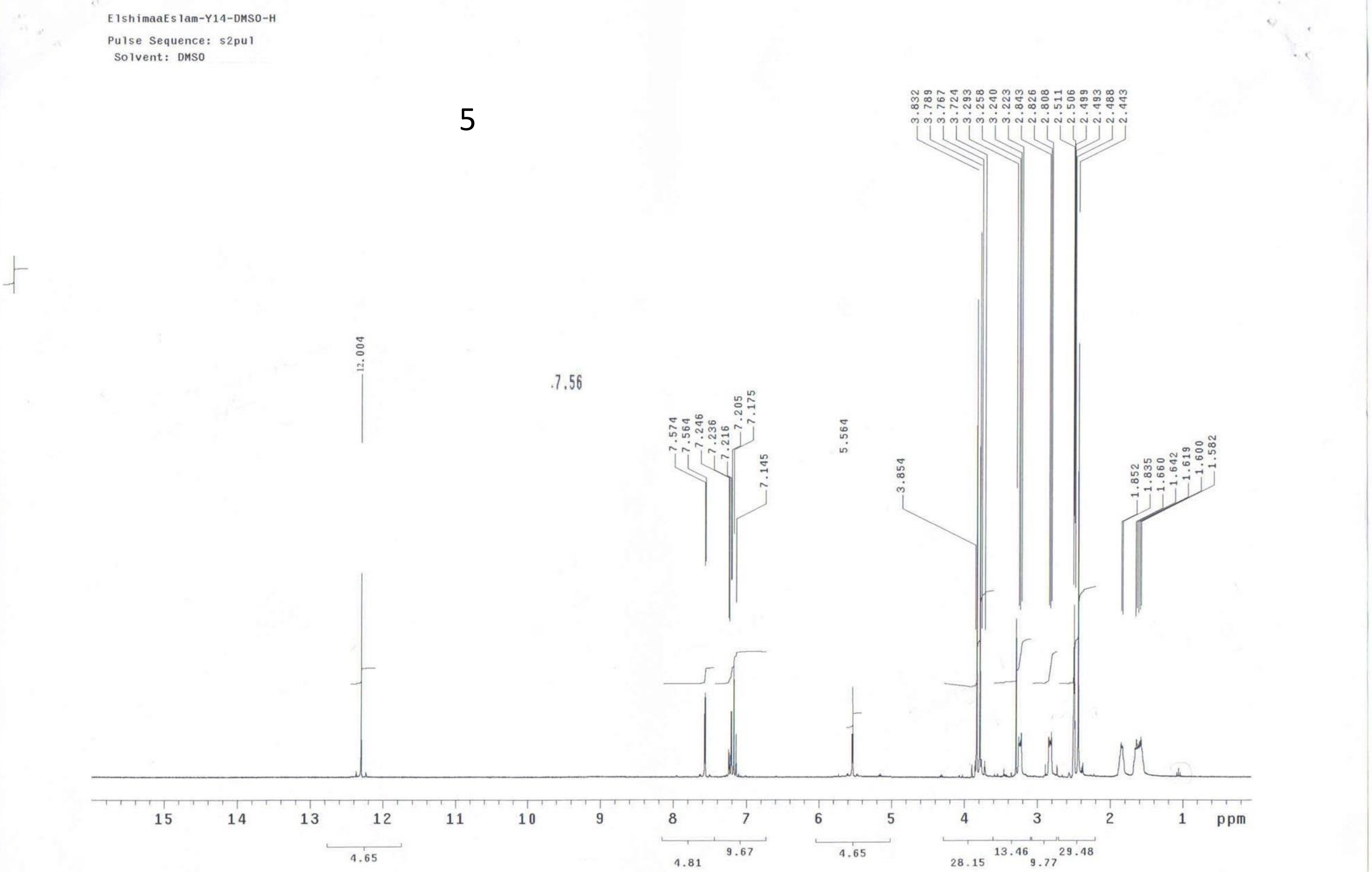
ElshimaaEslam-Y14-DMSO-H
Pulse Sequence: s2pul
Solvent: DMSO
12.004
7.56
7.574
7.564
7.246
7.236
7.216
7.205
7.175
7.145
5.564
3.854
3.832
3.789
3.767
3.724
3.293
3.258
3.240
3.223
2.843
2.826
2.808
2.511
2.506
2.499
2.493
2.488
2.443
1.852
1.835
1.660
1.642
1.619
1.600
1.582
15 14 13 12 11 10 9 8 7 6 5 4 3 2 1 ppm
4.65
4.81
9.67
4.65
28.15
13.46
9.77
29.48

ElshimaaEslam-T8-DMSO-D2O-H

Pulse Sequence: s2pul

Solvent: DMSO

6

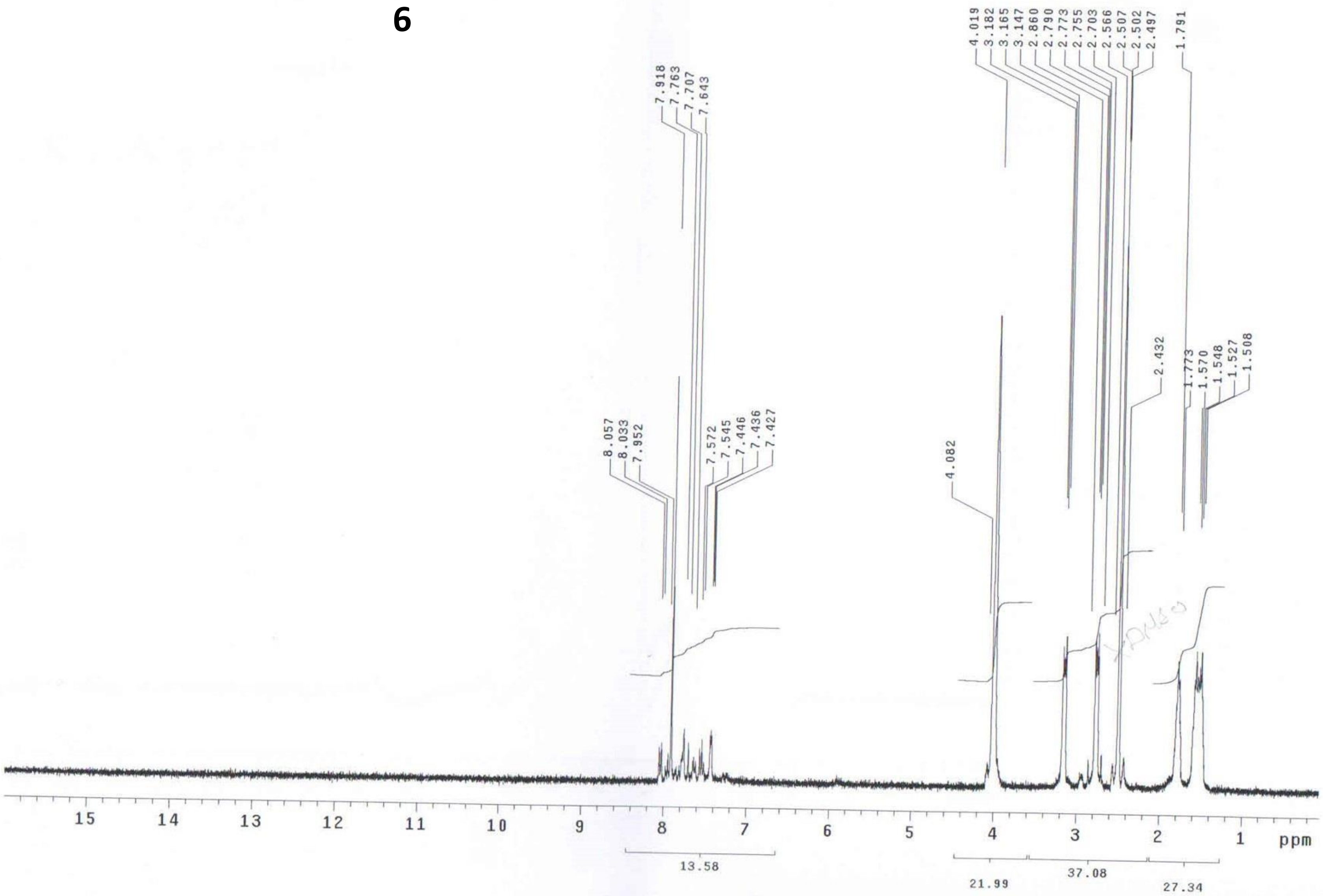

PROTON DMSO {C:\Bruker\TOPSPIN\KFUCCP} nmr 11

7b

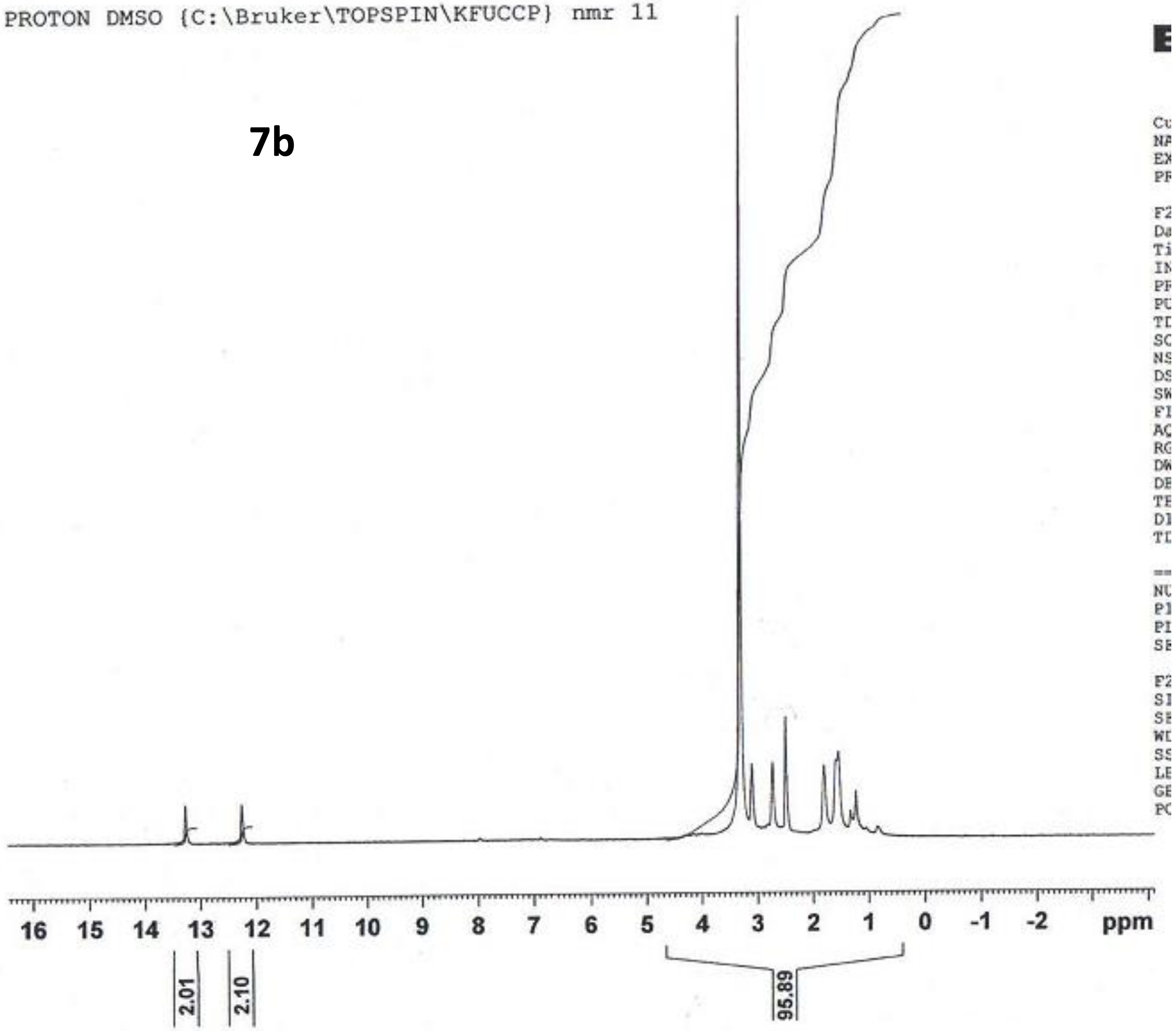

ElshimaaEslam-T7-DMSO-H
Pulse Sequence: s2pul
Solvent: DMSO
Temp. 30.0 C / 303.1 K

9b

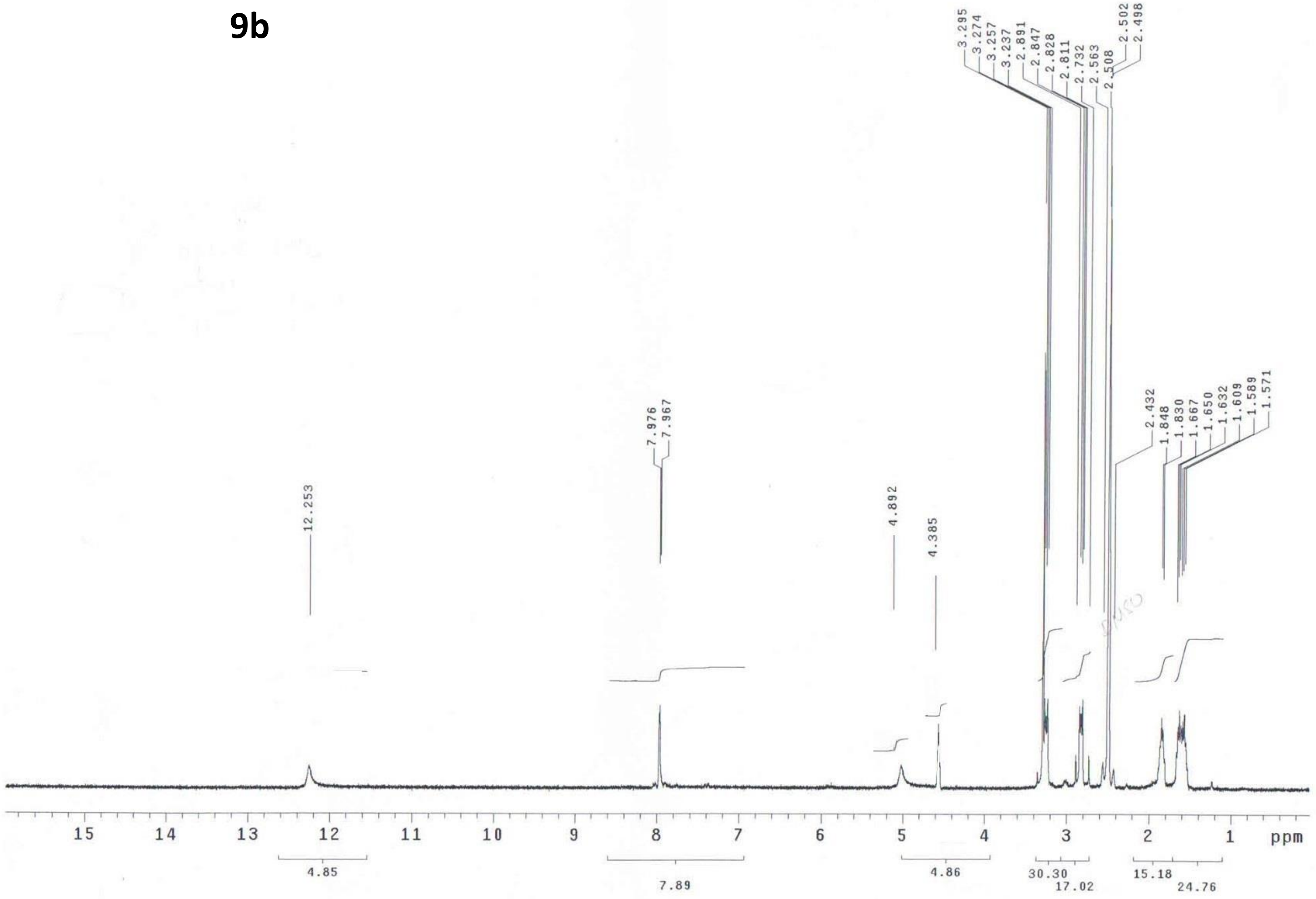

10a

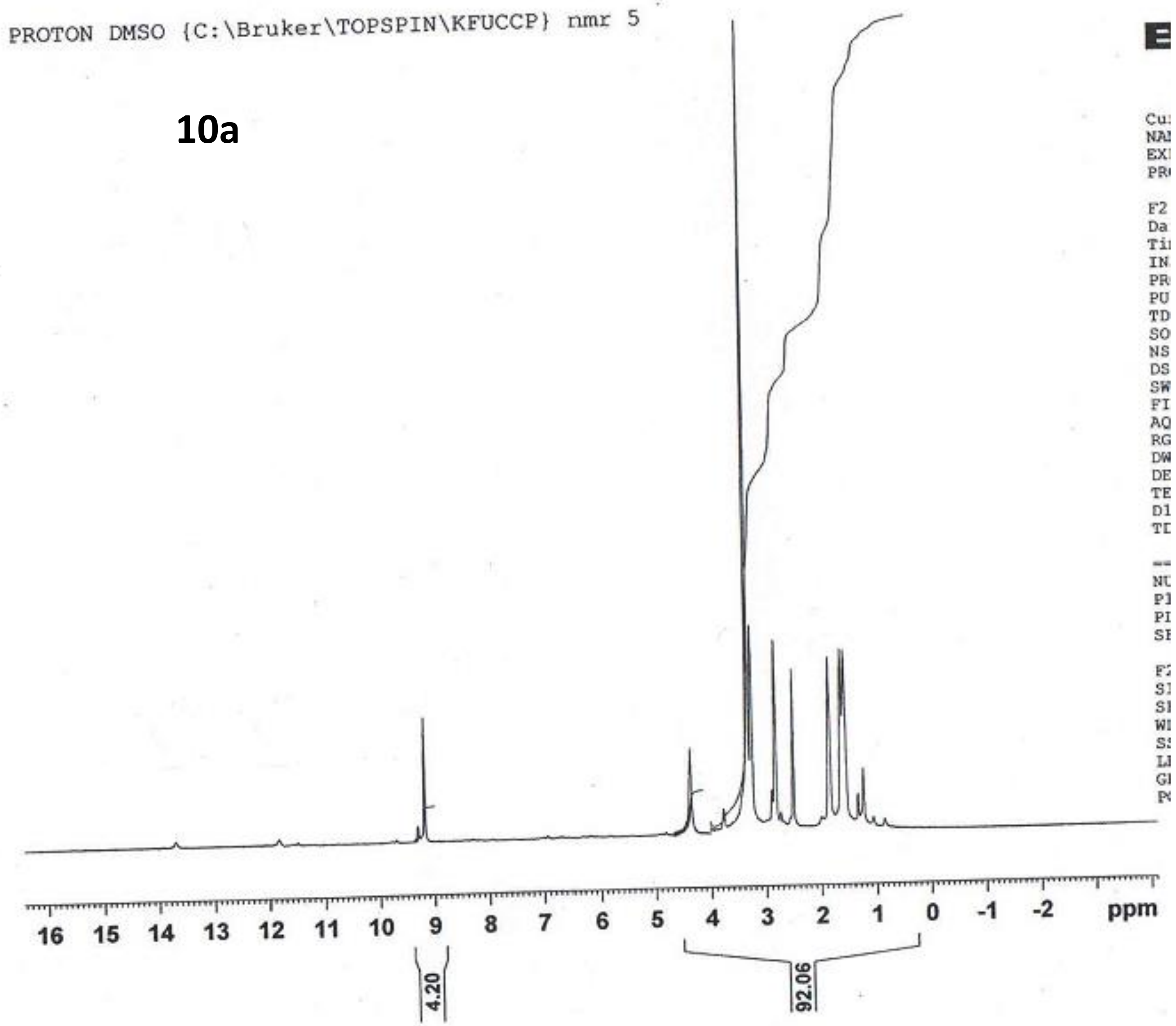
PROTON DMSO {C:\Bruker\TOPSPIN\KFUCCP} nmr 5
16 15 14 13 12 11 10 9 8 7 6 5 4 3 2 1 0 -1 -2 ppm
4.20
92.06
Cu
NA
EX
PR
F2
Da
Ti
IN
PR
PU
TD
SO
NS
DS
SW
FI
AQ
RG
DW
DE
TE
D1
TD
NU
P1
PL
SF
SI
SF
WD
SS
LB
GB
PC

ElshimaaEslam-YB-DMSO-H

Pulse Sequence: s2pul

Solvent: DMSO

11

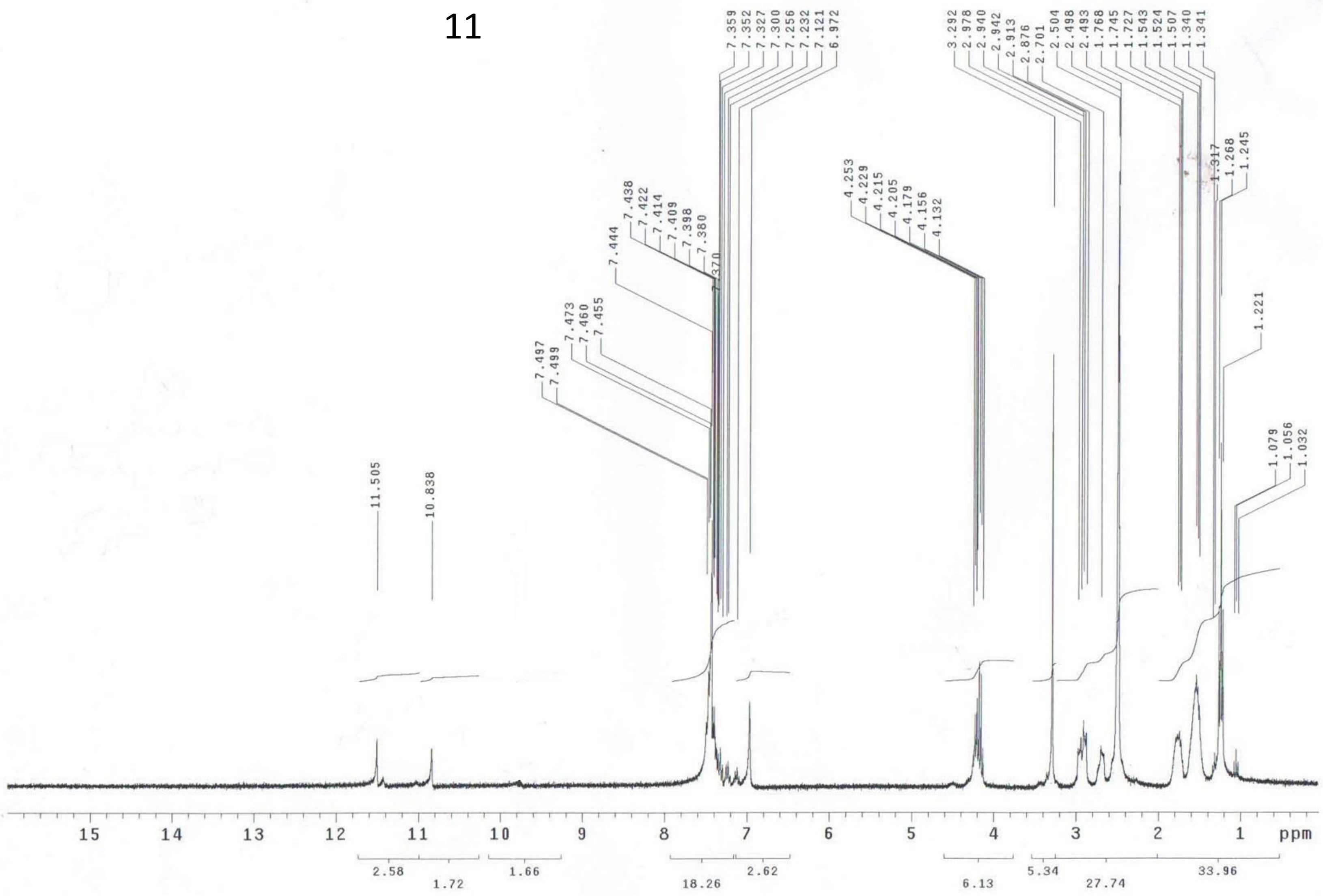

12

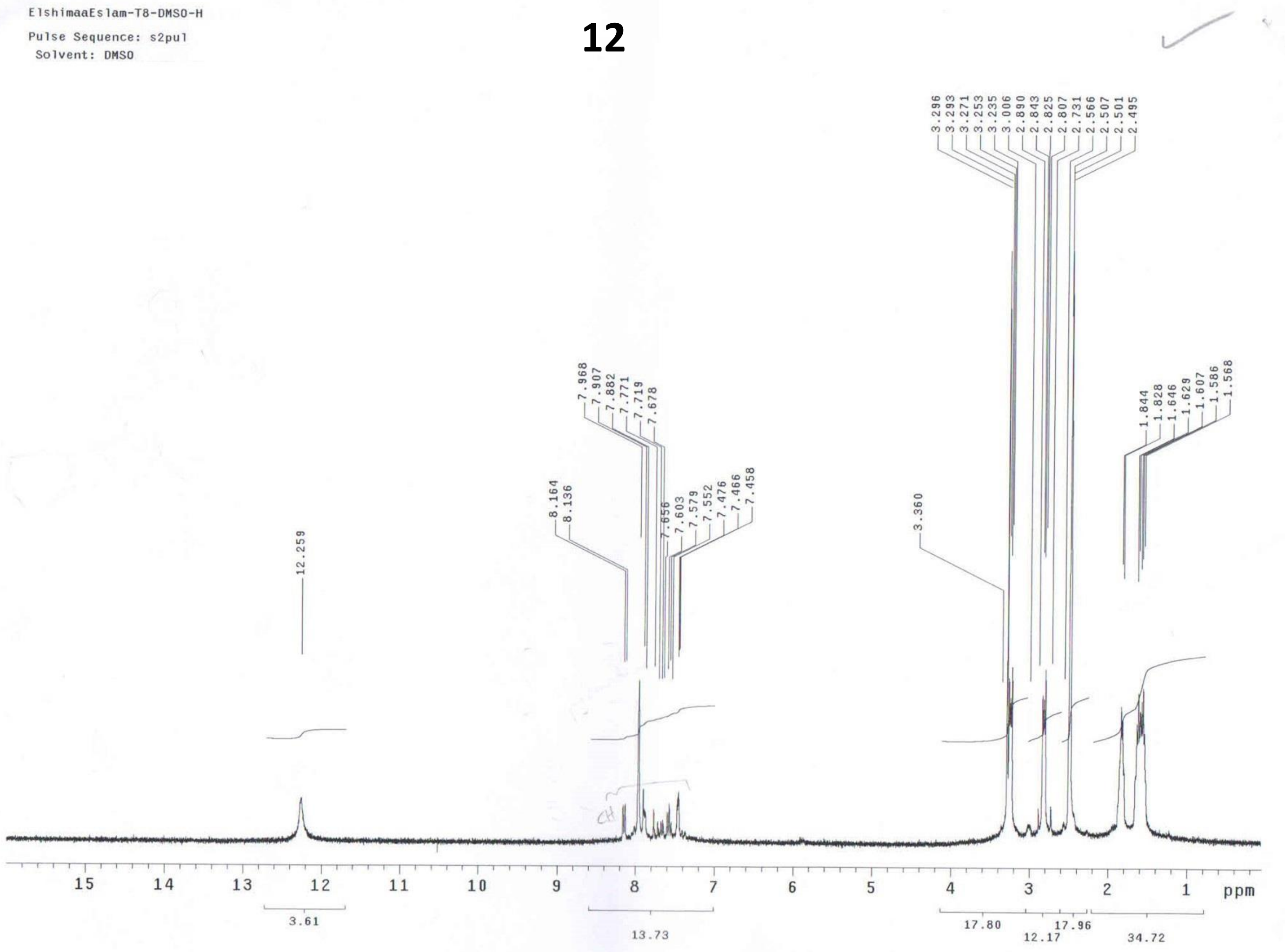
ElshimaaEslam-T8-DMSO-H
Pulse Sequence: s2pul
Solvent: DMSO
12.259
8.164
8.136
7.968
7.907
7.882
7.771
7.719
7.678
7.656
7.603
7.579
7.552
7.476
7.466
7.458
3.360
3.296
3.293
3.271
3.253
3.235
3.006
2.890
2.843
2.825
2.807
2.731
2.566
2.507
2.501
2.495
1.844
1.828
1.646
1.629
1.607
1.586
1.568
ppm
3.61
13.73
17.80
12.17
17.96
34.72

C-13-NMR

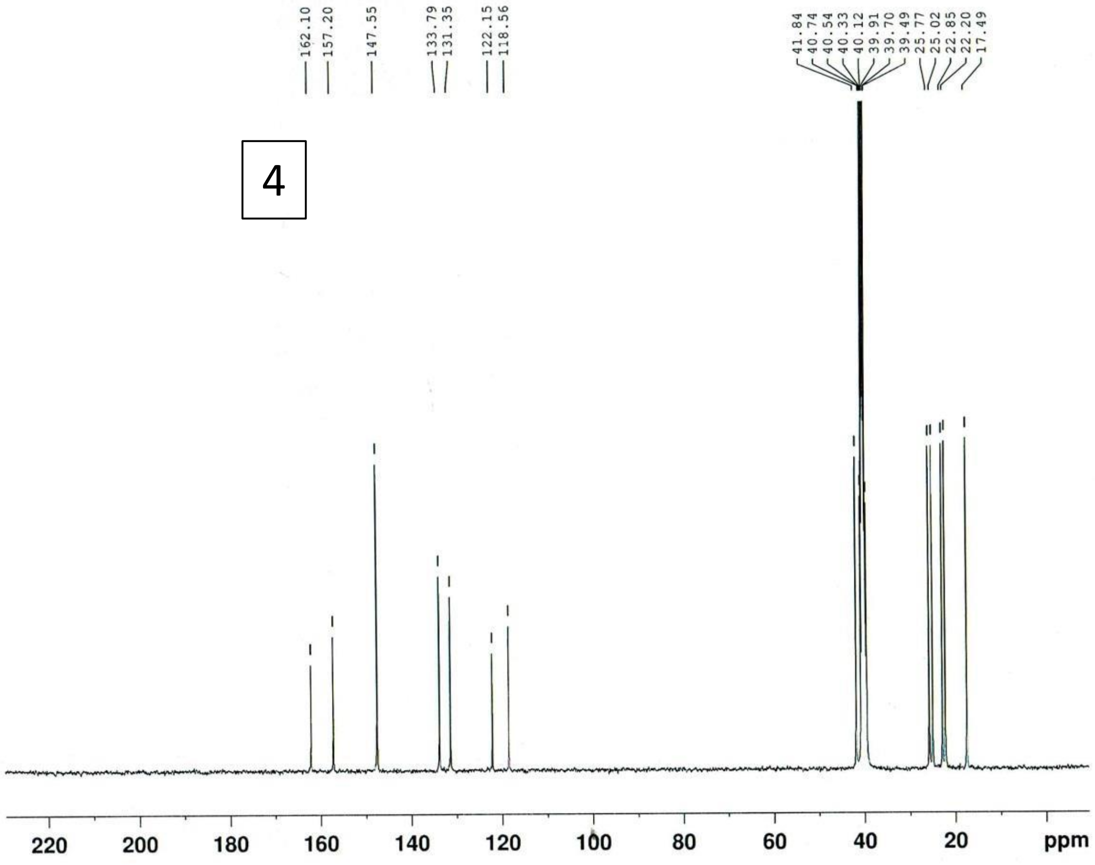
4
162.10
157.20
147.55
133.79
131.35
122.15
118.56
41.84
40.74
40.54
40.33
40.12
39.91
39.70
39.49
25.77
25.02
22.85
22.20
17.49
220
200
180
160
140
120
100
80
60
40
20
ppm

5

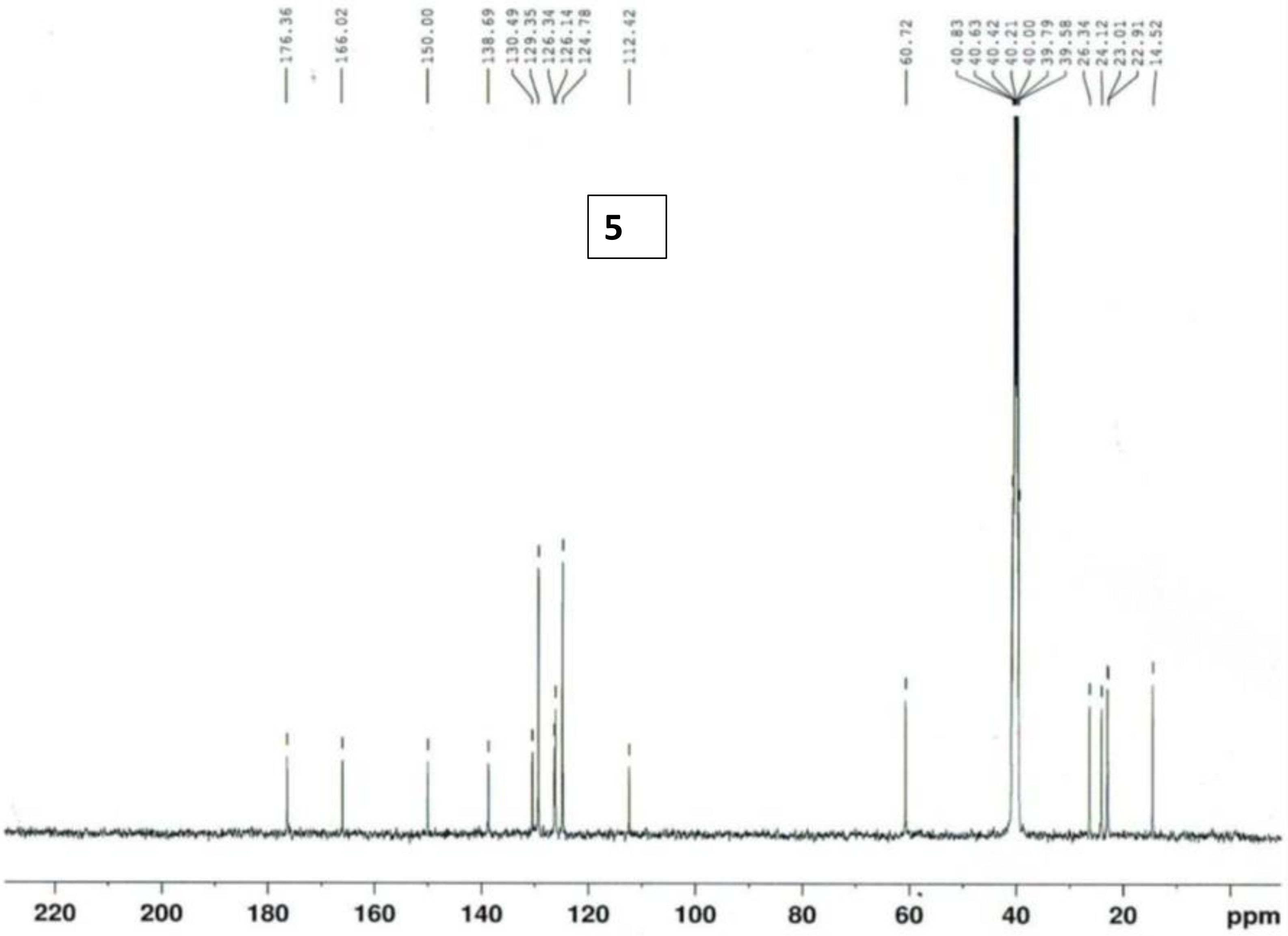
176.36
166.02
150.00
138.69
130.49
129.35
126.34
126.14
124.78
112.42
60.72
40.83
40.63
40.42
40.21
40.00
39.79
39.58
26.34
24.12
23.01
22.91
14.52
220
200
180
160
140
120
100
80
60
40
20
ppm

6

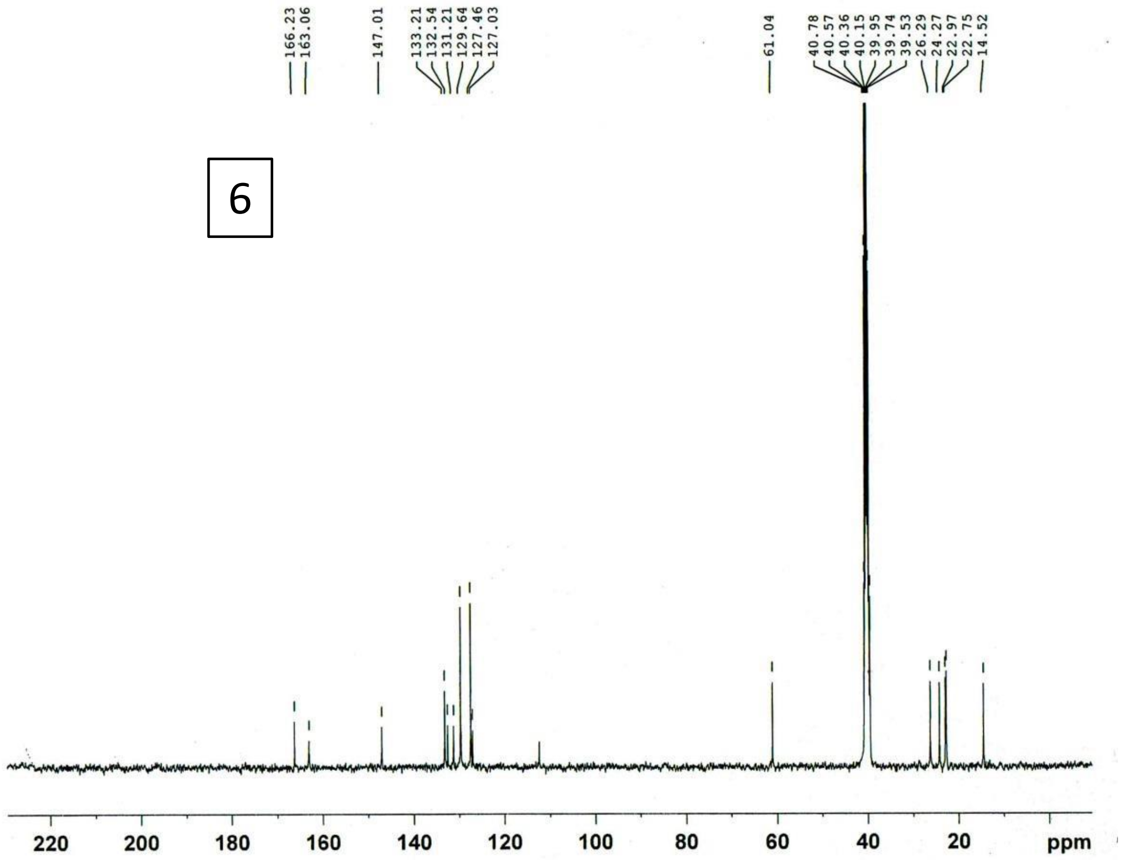
166.23
163.06
147.01
133.21
132.54
131.21
129.64
127.46
127.03
61.04
40.78
40.57
40.36
40.15
39.95
39.74
39.53
26.29
24.27
22.97
22.75
14.52
220 200 180 160 140 120 100 80 60 40 20 ppm

7

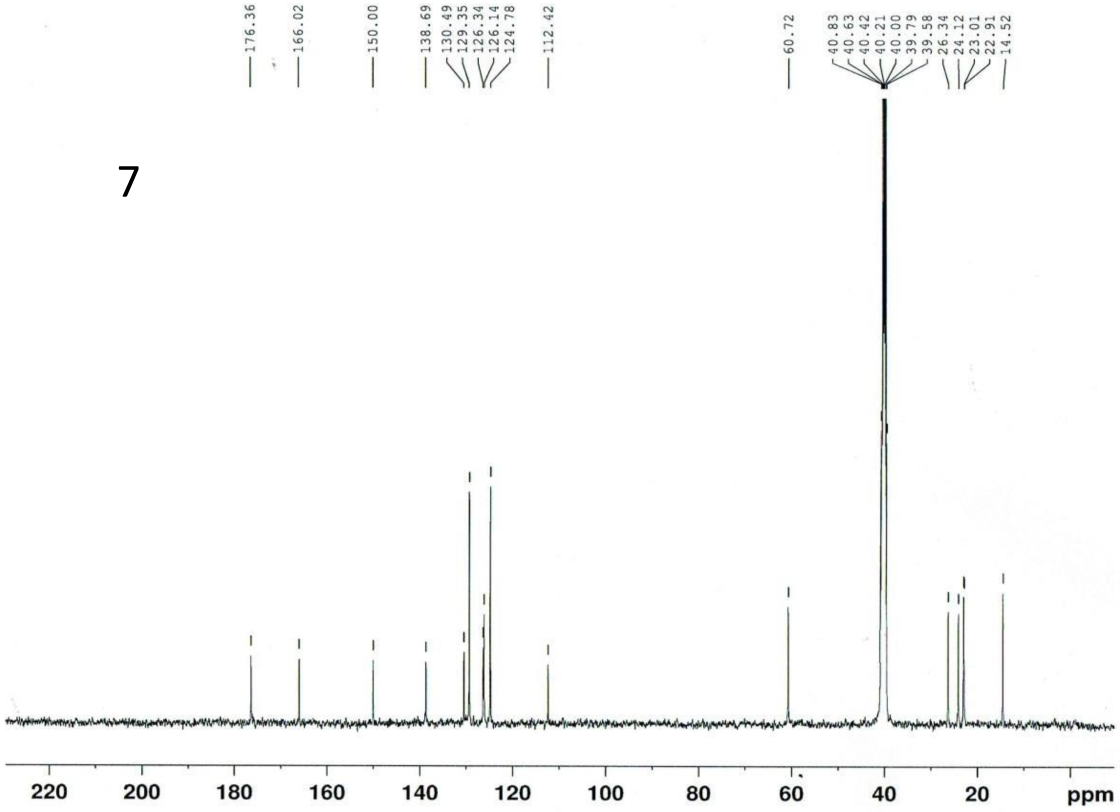

8

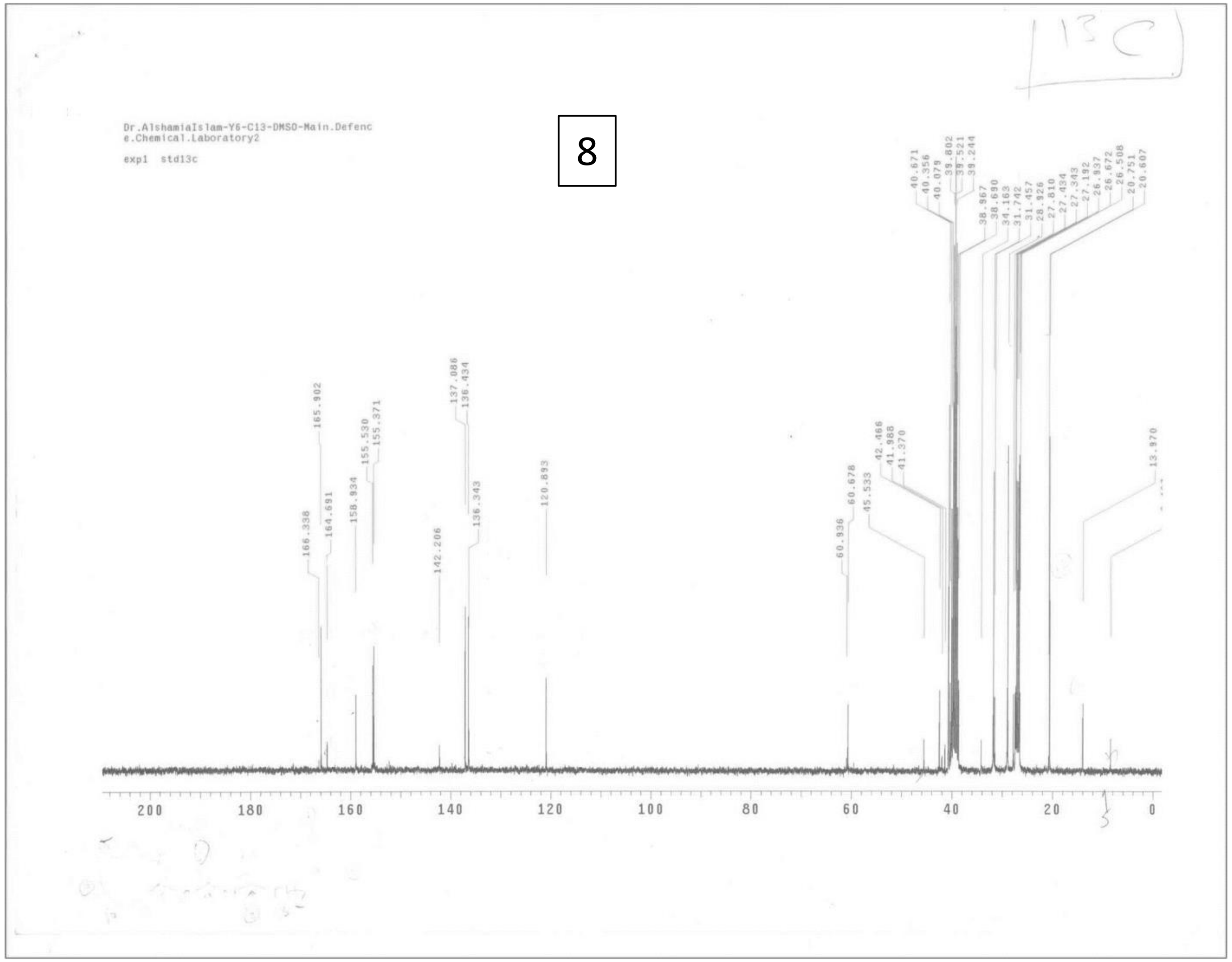
Dr.AlshamiaIslam-Y6-C13-DMSO-Main.Defenc
e.Chemical.Laboratory2
exp1 std13c
13C
166.338
165.902
164.691
158.934
155.530
155.371
142.206
137.086
136.434
136.343
120.893
60.936
60.678
45.533
42.466
41.988
41.370
40.671
40.356
40.079
39.802
39.521
39.244
38.967
38.690
34.163
31.742
31.457
28.926
27.810
27.434
27.343
27.192
26.937
26.672
26.508
20.751
20.607
13.970
200
180
160
140
120
100
80
60
40
20
0

9

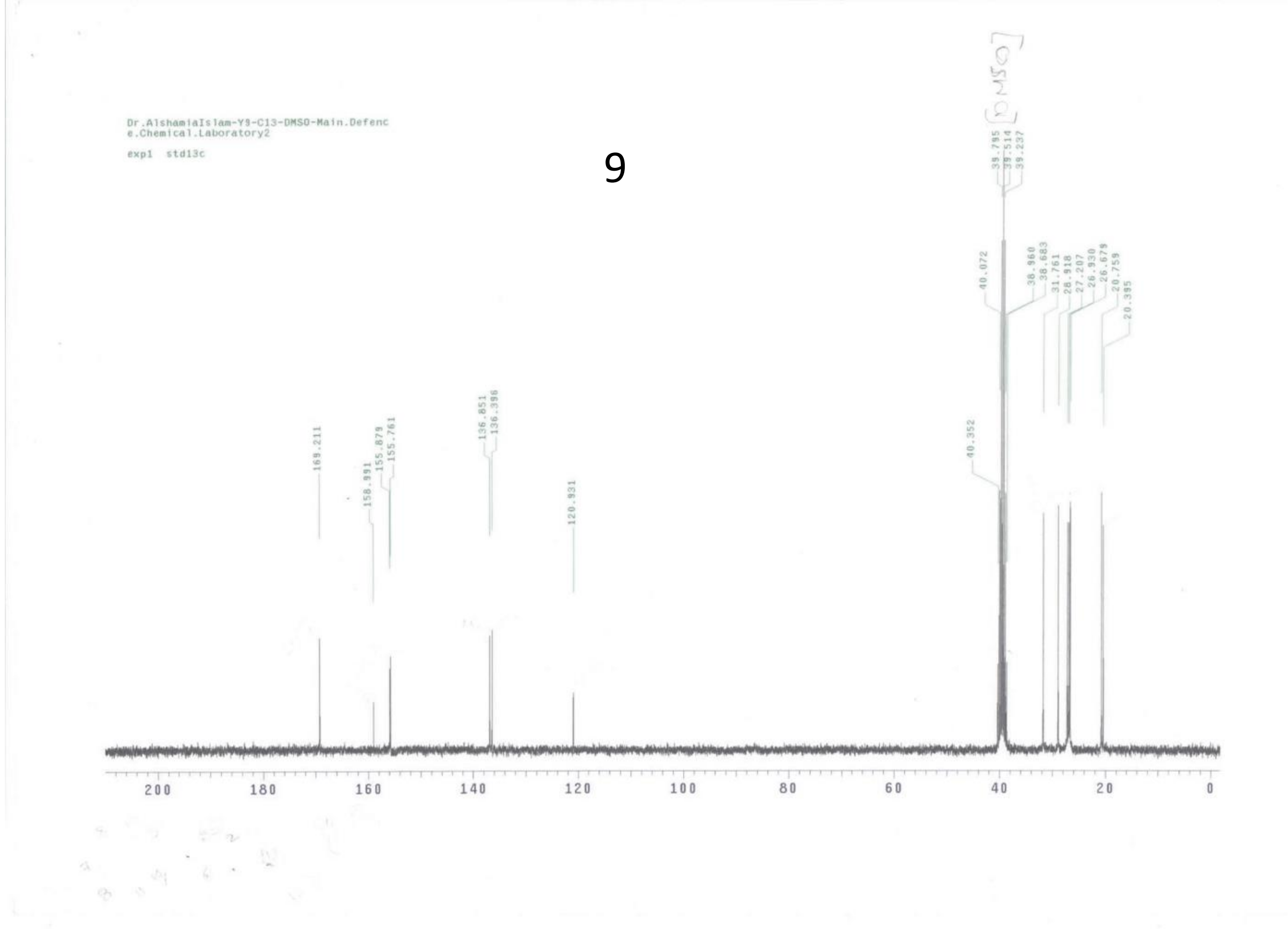
Dr.AlshamiaIslam-Y9-C13-DMSO-Main.Defence.Chemical.Laboratory2
exp1 std13c
169.211
158.991
155.879
155.761
136.851
136.396
120.931
40.352
40.072
39.795
39.514
39.237
38.960
38.683
31.761
28.918
27.207
26.930
26.679
26.759
20.395
DMSO
200
180
160
140
120
100
80
60
40
20
0

10

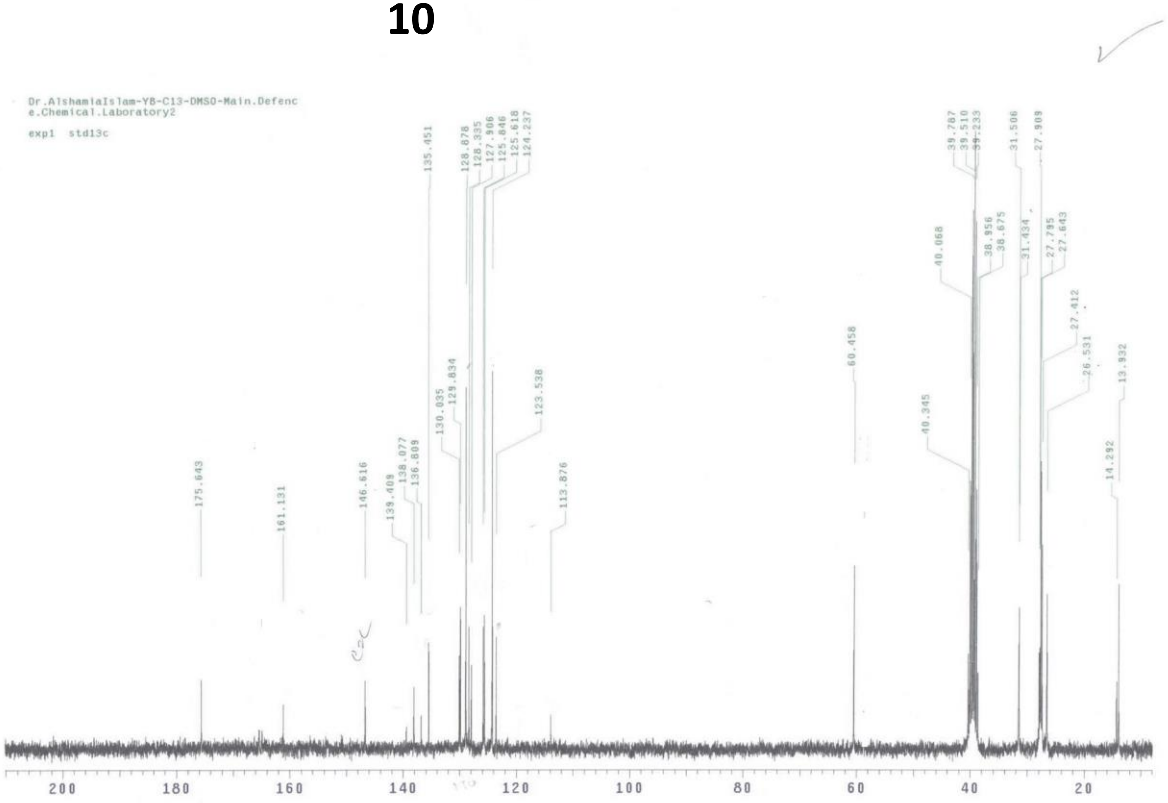
Dr.AlshamiaIslam-Y8-C13-DMSO-Main.Defenc
e.Chemical.Laboratory2
exp1 std13c
175.643
161.131
146.616
139.409
138.077
136.809
135.451
130.035
129.834
128.878
128.335
127.906
125.846
125.618
124.237
123.538
113.876
60.458
40.345
40.068
39.787
39.510
39.233
38.956
38.675
31.506
31.434
27.909
27.795
27.643
27.412
26.531
14.292
13.932
200
180
160
140
120
100
80
60
40
20

11

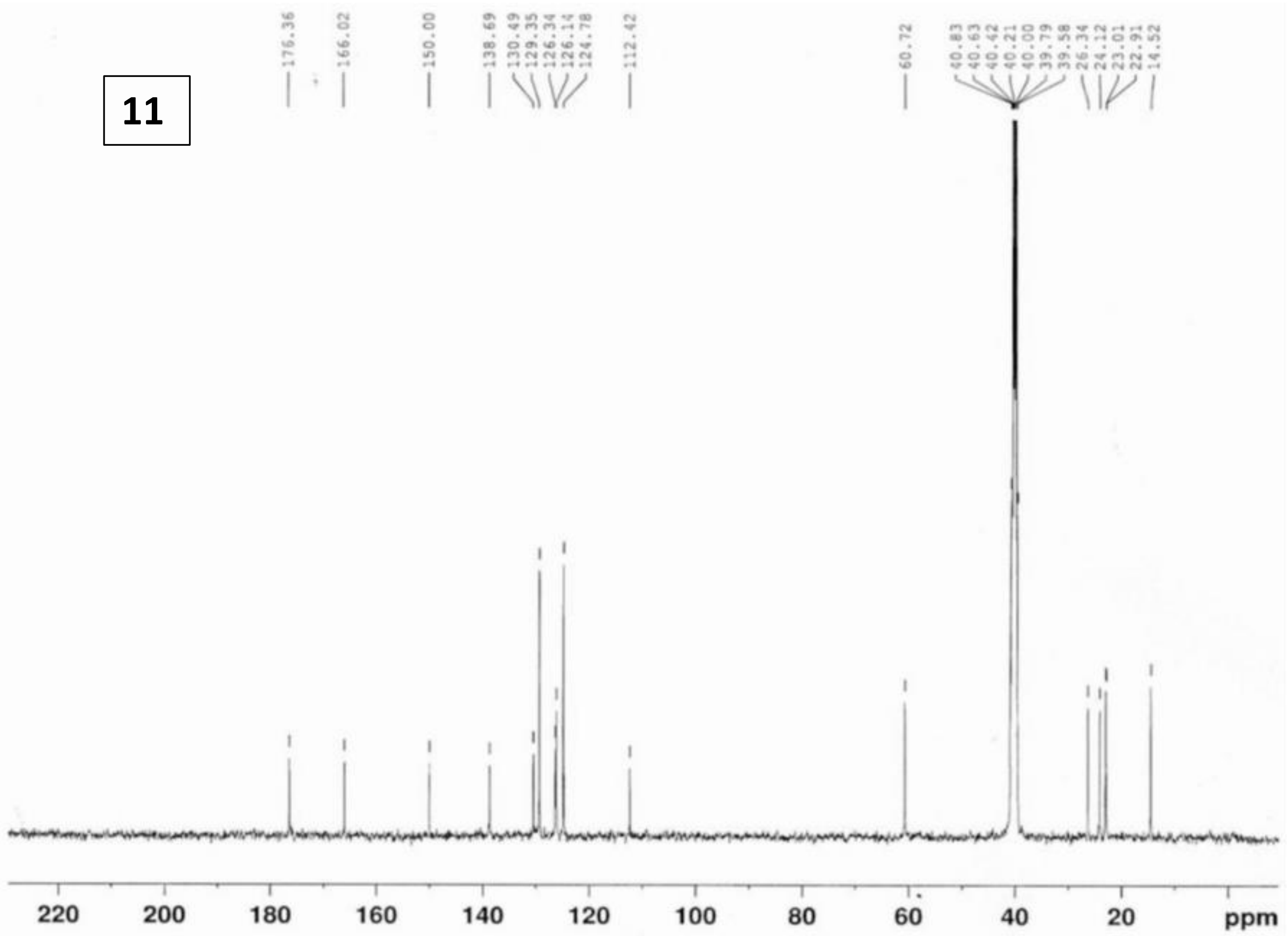
176.36
166.02
150.00
138.69
130.49
129.35
126.34
126.14
124.78
112.42
60.72
40.83
40.63
40.42
40.21
40.00
39.79
39.58
26.34
24.12
23.01
22.91
14.52
220
200
180
160
140
120
100
80
60
40
20
ppm

12

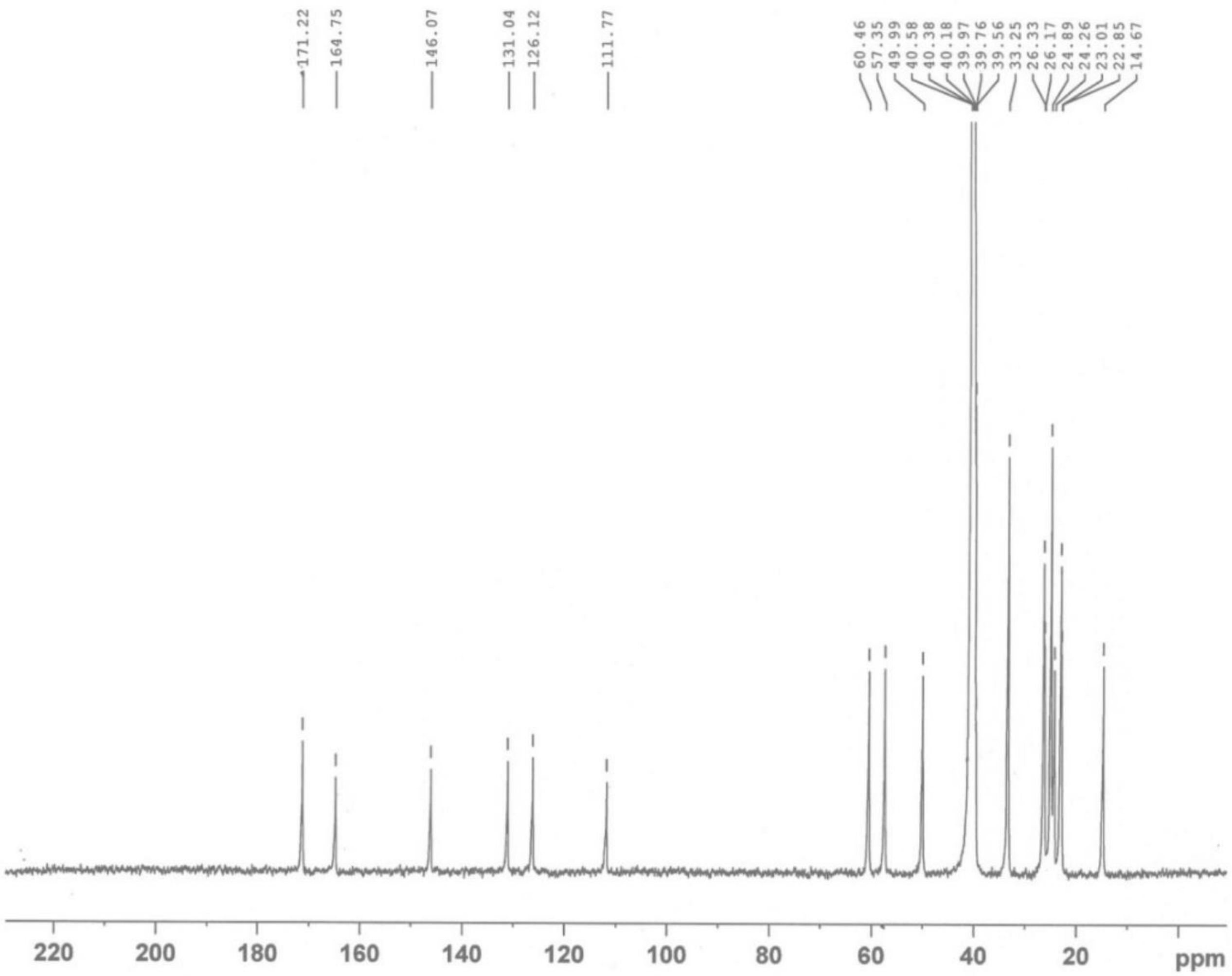
171.22
164.75
146.07
131.04
126.12
111.77
60.46
57.35
49.99
40.58
40.38
40.18
39.97
39.76
39.56
33.25
26.33
26.17
24.89
24.26
23.01
22.85
14.67
220
200
180
160
140
120
100
80
60
40
20
ppm

Target prediction

9a

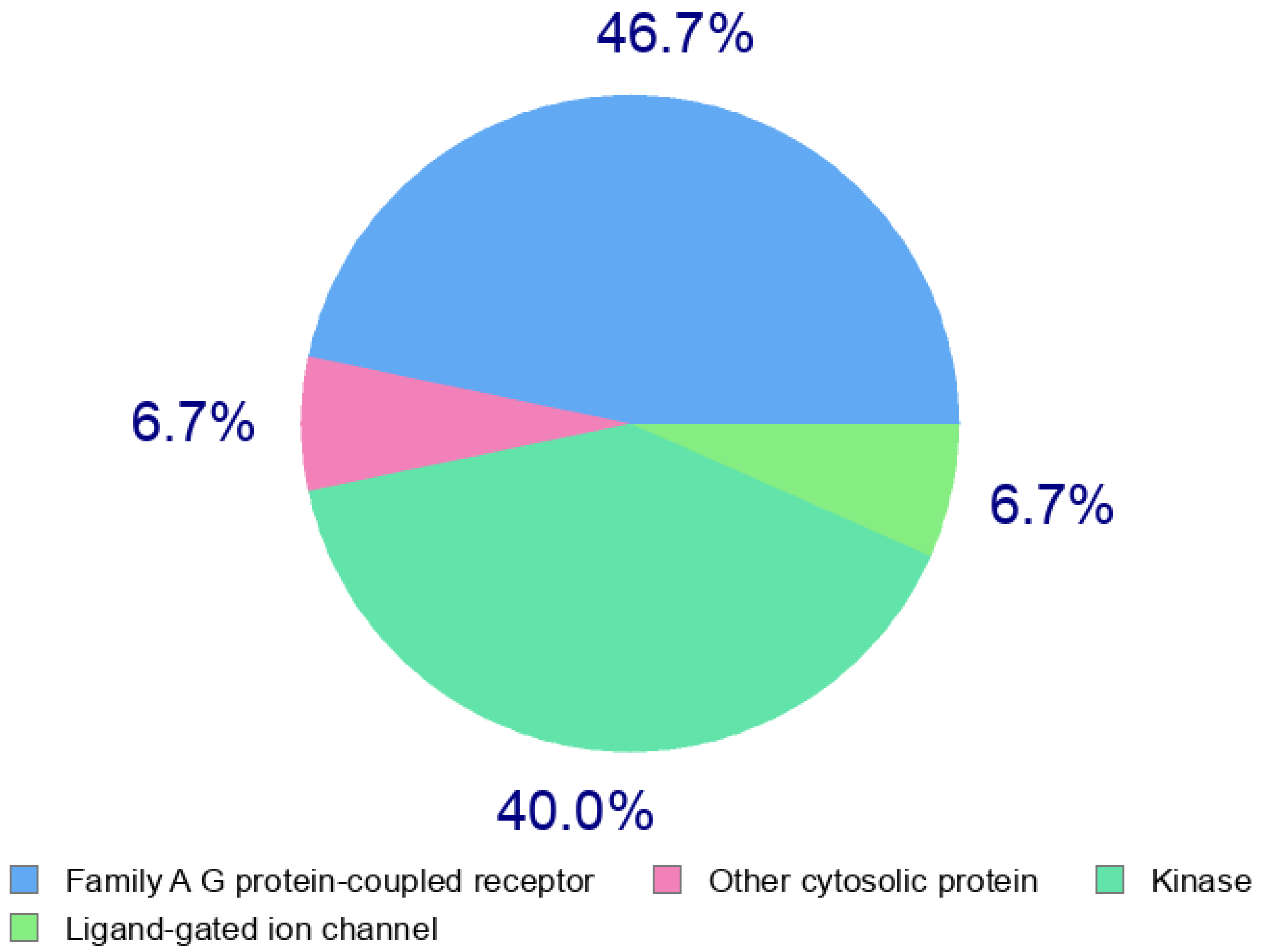
46.7%
6.7%
6.7%
40.0%
Family A G protein-coupled receptor
Other cytosolic protein
Kinase
Ligand-gated ion channel

9b

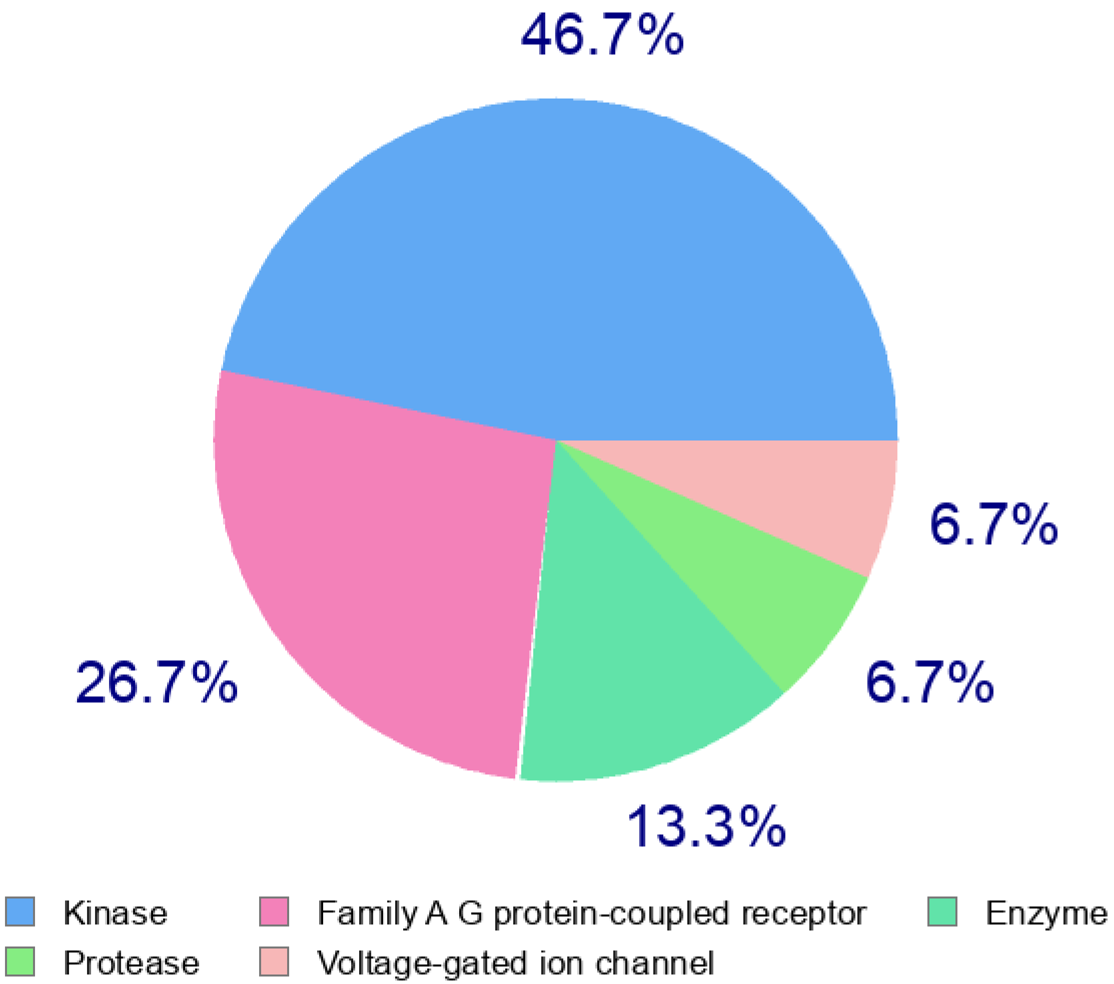
46.7%
6.7%
6.7%
13.3%
26.7%
Kinase
Family A G protein-coupled receptor
Enzyme
Protease
Voltage-gated ion channel

10a

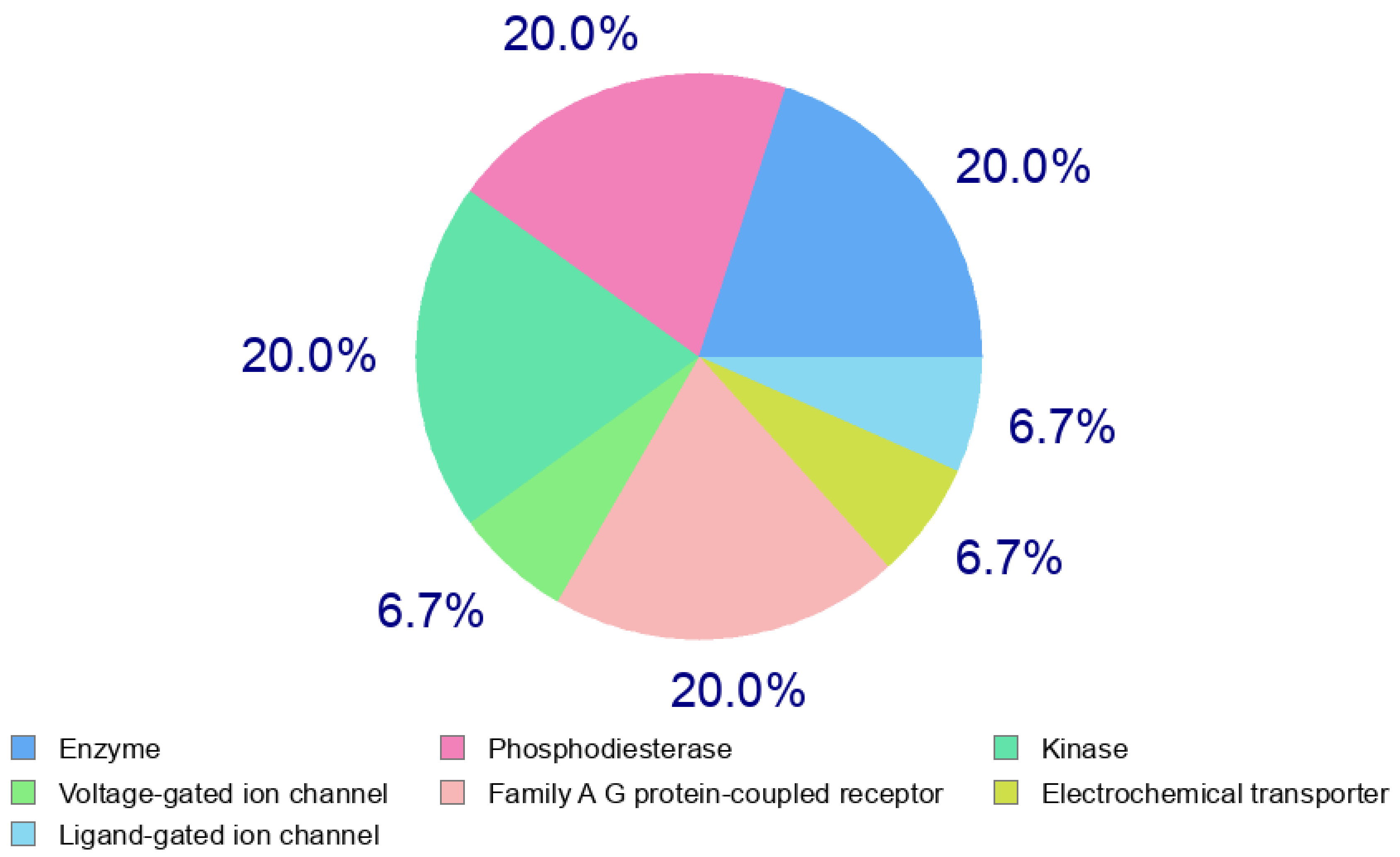
20.0%
20.0%
20.0%
6.7%
6.7%
6.7%
20.0%
Enzyme
Phosphodiesterase
Kinase
Voltage-gated ion channel
Family A G protein-coupled receptor
Electrochemical transporter
Ligand-gated ion channel

10b

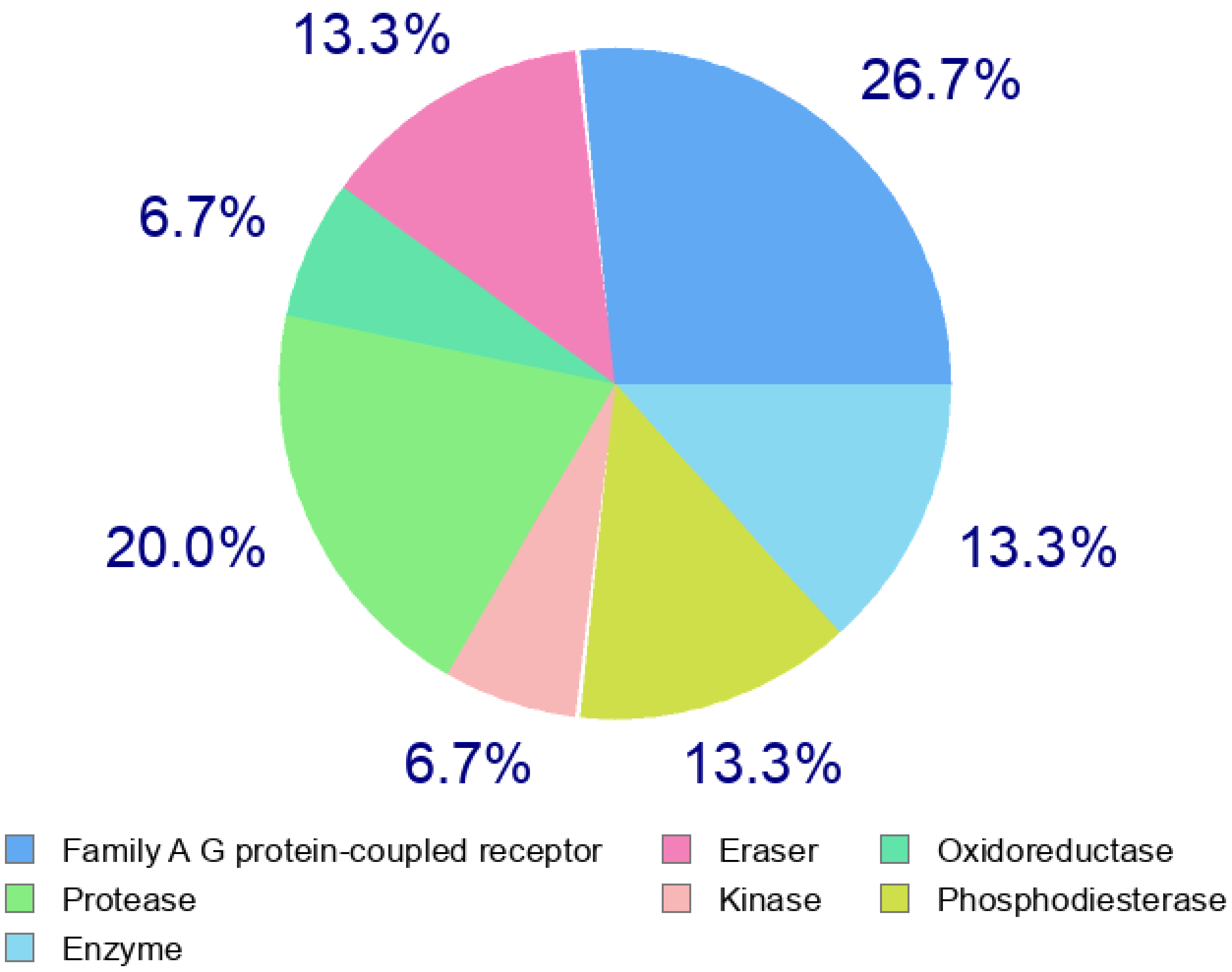
13.3%
26.7%
6.7%
20.0%
13.3%
6.7%
13.3%
Family A G protein-coupled receptor
Eraser
Oxidoreductase
Protease
Kinase
Phosphodiesterase
Enzyme

12

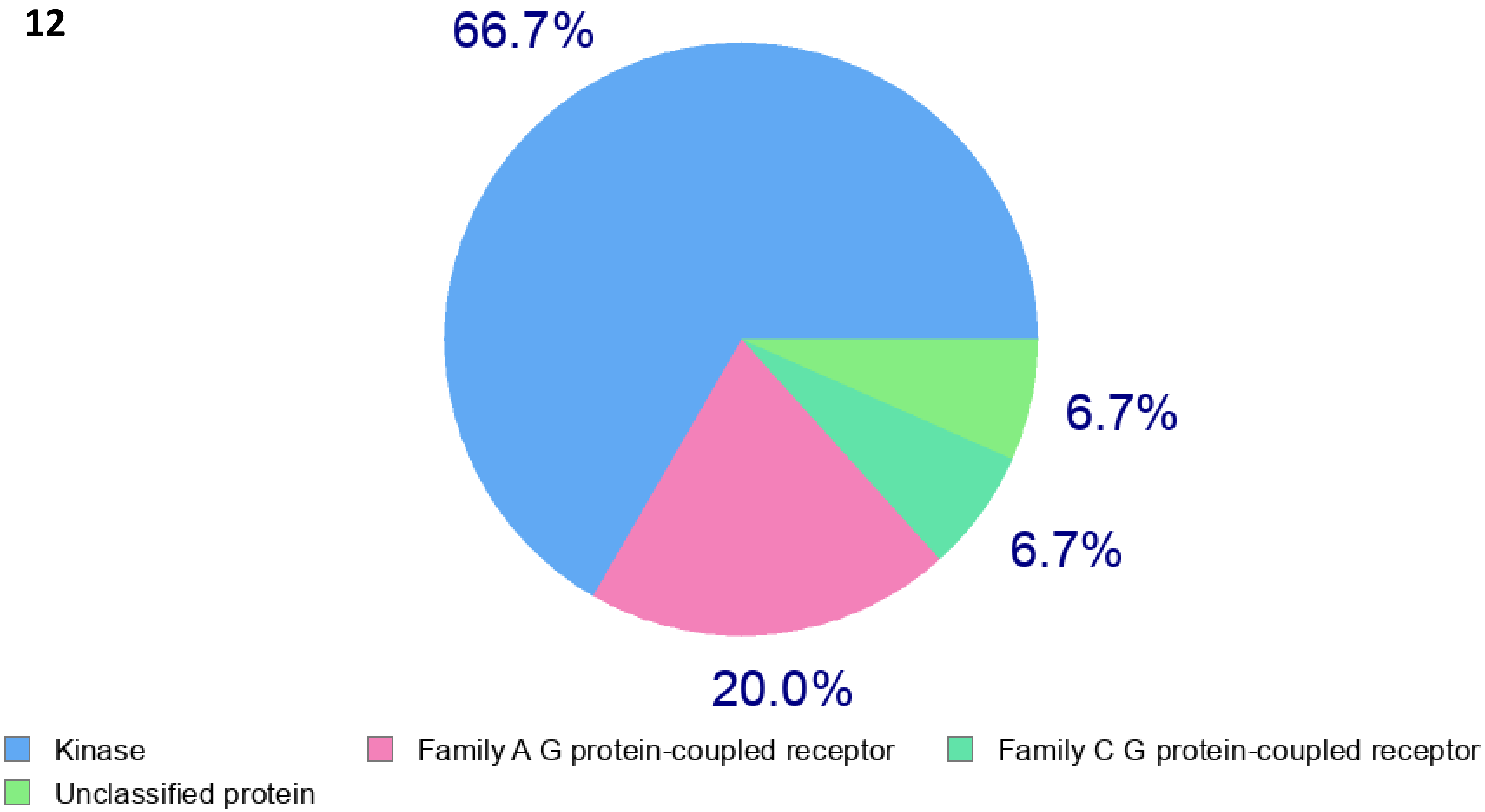
66.7%
6.7%
6.7%
20.0%
Kinase
Family A G protein-coupled receptor
Family C G protein-coupled receptor
Unclassified protein

8

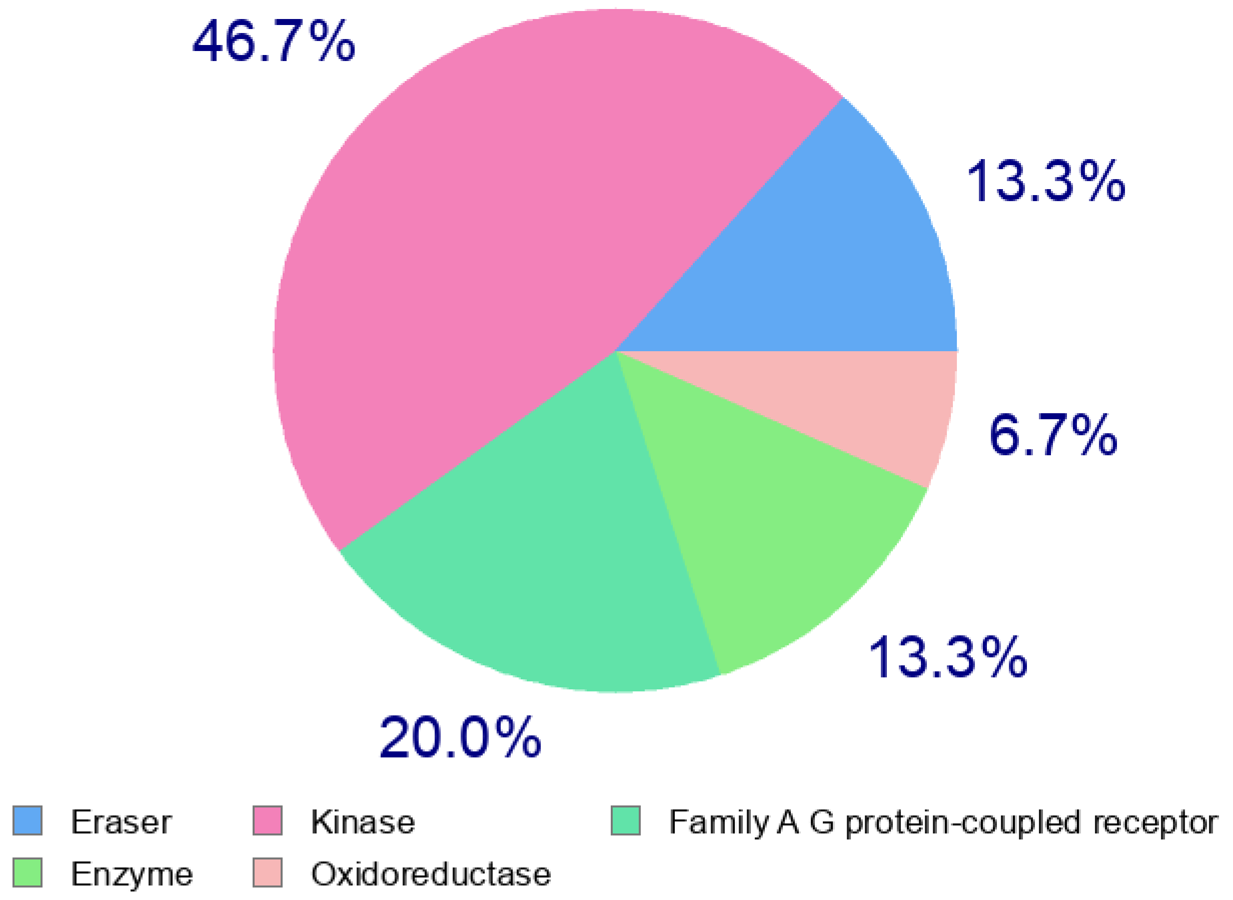
46.7%
13.3%
6.7%
13.3%
20.0%
Eraser
Kinase
Family A G protein-coupled receptor
Enzyme
Oxidoreductase
